# Telomerase-Null Survivor Screening Identifies Novel Telomere Recombination Regulators

**DOI:** 10.1371/journal.pgen.1003208

**Published:** 2013-01-17

**Authors:** Yan Hu, Hong-Bo Tang, Ning-Ning Liu, Xia-Jing Tong, Wei Dang, Yi-Min Duan, Xiao-Hong Fu, Yang Zhang, Jing Peng, Fei-Long Meng, Jin-Qiu Zhou

**Affiliations:** The State Key Laboratory of Molecular Biology, Shanghai Institute of Biochemistry and Cell Biology, Shanghai Institutes for Biological Sciences, Chinese Academy of Sciences, University of Chinese Academy of Sciences, Shanghai, China; University of Georgia, United States of America

## Abstract

Telomeres are protein–DNA structures found at the ends of linear chromosomes and are crucial for genome integrity. Telomeric DNA length is primarily maintained by the enzyme telomerase. Cells lacking telomerase will undergo senescence when telomeres become critically short. In *Saccharomyces cerevisiae*, a very small percentage of cells lacking telomerase can remain viable by lengthening telomeres via two distinct homologous recombination pathways. These “survivor” cells are classified as either Type I or Type II, with each class of survivor possessing distinct telomeric DNA structures and genetic requirements. To elucidate the regulatory pathways contributing to survivor generation, we knocked out the telomerase RNA gene *TLC1* in 280 telomere-length-maintenance (TLM) gene mutants and examined telomere structures in post-senescent survivors. We uncovered new functional roles for 10 genes that affect the emerging ratio of Type I versus Type II survivors and 22 genes that are required for Type II survivor generation. We further verified that Pif1 helicase was required for Type I recombination and that the INO80 chromatin remodeling complex greatly affected the emerging frequency of Type I survivors. Finally, we found the Rad6-mediated ubiquitination pathway and the KEOPS complex were required for Type II recombination. Our data provide an independent line of evidence supporting the idea that these genes play important roles in telomere dynamics.

## Author Summary

Homologous recombination is a means for an organism or a cell to repair damaged DNA in its genome. Eukaryotic chromosomes have a linear configuration with two ends that are special DNA–protein structures called telomeres. Telomeres can be recognized by the cell as DNA double-strand breaks and subjected to repair by homologous recombination. In the baker's yeast *Saccharomyces cerevisiae*, cells that lack the enzyme telomerase, which is the primary factor responsible for telomeric DNA elongation, are able to escape senescence and cell death when telomeres undergo repair via homologous recombination. In this study, we have performed genetic screens to identify genes that affect telomeric DNA recombination. By examining the telomere structures in 280 mutants, each of which lacks both a telomere-length-maintenance gene and telomerase RNA gene, we identified 32 genes that were not previously known to be involved in telomere recombination. These genes have functions in a variety of cellular processes, and our work provides new insights into the regulation of telomere recombination in the absence of telomerase.

## Introduction

Telomeres are special DNA-protein structures found at the ends of eukaryotic chromosomes. Telomeres are crucial for genome integrity because they prevent chromosome ends from degradation or fusing with each other [1]. In budding yeast *Saccharomyces cerevisiae*, telomeric DNA consists of ∼350 base pairs (bp) of TG_1–3_/C_1–3_ A repeats with a terminal single-stranded TG_1–3_ tract called a G-overhang [2]. Telomeric DNA can be maintained by either telomerase-mediated elongation or homologous recombination [3]–[5]. Telomerase is a highly specialized reverse transcriptase that adds telomeric DNA sequences to the 3′ G-overhang using its intrinsic RNA template [3]. In *Saccharomyces cerevisiae*, the core components of telomerase are the catalytic subunit Est2 and its RNA template subunit TLC1 [6], [7]. In wild-type yeast cells, the telomerase pathway supercedes the recombination pathway as the predominant mechanism of telomeric DNA elongation [8], [9]. In telomerase-null cells, telomeric DNA is maintained via a recombination pathway termed “alternative lengthening of telomeres” (ALT) [10]. Approximately 85% of immortalized human tumor cells use telomerase to maintain telomeres while 15% apply the ALT mechanism to maintain telomeres [11].

In telomerase-null *S. cerevisiae* mutants, most cells undergo senescence after about 50–100 divisions when telomeres shorten to less than approximately 100 bp [7], [12], [13]. Surprisingly, a select few of these senescing cells are able to bypass the short telomere survival crisis through lengthening their telomeres via a Rad52-dependent recombination pathway [14]. These cells are called post-senescence survivors or “survivors” for short [14]. Survivors are categorized into two types: Type I and Type II, which possess different telomeric DNA structures and are defined by their dependence on Rad51 or Rad50 respectively [15]. Type I survivors exhibit highly amplified subtelomeric Y' elements and short terminal telomeric TG tracts. The formation of Type I survivors depends on the canonical homologous recombination proteins Rad51, Rad54, Rad55 and Rad57 [14]. On the other hand, Type II survivors have long heterogeneous terminal telomeric TG tracts generated by recombination, and their formation depends on the Mre11-Rad50-Xrs2 (MRX) complex and Rad59 [14]. Type II survivors resemble the ALT cells observed in mammals [5]. In *S. cerevisiae*, about 90% of survivors generated on solid medium are categorized as Type I, while 10% are Type II. Nevertheless, Type II survivors grow at faster rates than Type I survivors, eventually overtaking their counterparts in liquid-grown cultures [14].

In addition to the proteins in the Rad52 epistasis group, which are well-defined in the canonical survivor formation pathways, other genes involved in survivor formation have sporadically been identified. For example, *SGS1*, *MEC1*/*TEL1*, *MDT1*, *DEF1*, *CLB2* and *SUA5* are required for the generation of Type II survivors, while *RIF1* and *RIF2* have strong influences toward Type I survivor emerging frequency [16]–[22]. Notably, some of the genes mentioned above appear to contribute to both survivor generation and telomere length regulation. Deletion of *RIF1* or *RIF2* causes telomere lengthening, while deletion of *MRE11*, *RAD50*, *XRS2*, *TEL1*, *DEF1* or *SUA5* results in telomere shortening [16], [23]–[25]. These observations suggest that genes involved in telomere recombination pathways and telomere length regulation are in some way linked. So far, there have been 251 telomere length maintenance (TLM) genes identified by genome-wide screens [23], [26] and other studies [16], [27]–[34]. Furthermore, 29 additional genes previously miss-classified as essential genes in the *Saccharomyces* genome deletion project have now officially been implicated as TLM genes [24]. In this study we deleted the *TLC1* gene encoding the RNA template subunit of telomerase in each of these 280 TLM mutants. We then examined the survivor types that arose and in doing so we were able to identify novel regulators that contribute to telomere recombination. The genes we characterized as telomere recombination regulators may also affect general DNA recombination at other genomic loci.

## Results

### Screening of TLM gene deletion library on solid medium identifies genes affecting the emerging ratio of Type I versus Type II survivors

To search for genes affecting survivor formation, we knocked out the RNA component of telomerase *TLC1* in 280 haploid TLM mutants reported to have longer or shorter telomeres than the wild-type strain [23], [24], [26] (Table S1). Knocking out *TLC1* in most TLM mutants is typically achieved by transformation of an integrating plasmid but for some strains with extremely short telomeres or severe growth defects, recovering a *TLC1* deletion clone using this approach was not possible. For such cases, we mated *tlc1*Δ mutant (BY4741 background) with *tlm*Δ mutants (BY4742 background) to generate heterozygous diploid strains, and then performed tetrad dissection to obtain haploid mutants lacking both *TLC1* and TLM genes (Table S1).

**Figure 1:**
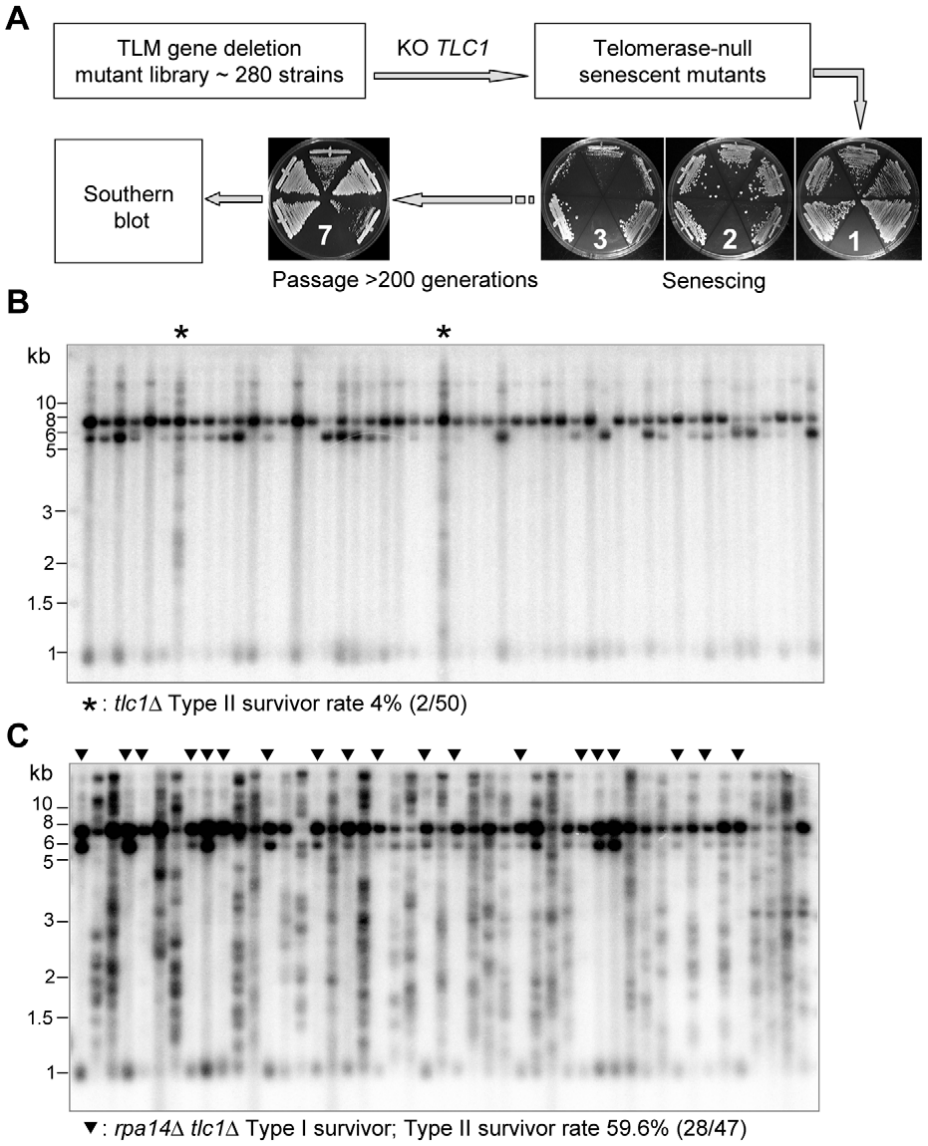
Identification of genes affecting the emerging ratio of Type I versus Type II survivors. (A) Schematic illustration of the screening procedures for genes that affect the emerging ratio of Type I vs Type II survivors (refer to details in main text). The *tlc1*Δ single mutants and the *rpa14*Δ *tlc1*Δ double mutants were generated through tetrad dissection from heterozygous diploids with one copy of *RPA14* and *TLC1* deleted. Fifty independent colonies of each mutant were randomly selected and passaged on plates, and the telomere structures of survivors were examined by Southern blot using a TG probe. (B and C) The Southern blot analysis of survivor types in *tlc1*Δ strain (control) (B) and the *rpa14*Δ *tlc1*Δ mutant (C). The asterisks (*) in (B) indicate Type II survivors. The triangles (▾) in (C) indicate Type I survivors.

After a telomerase-null *tlm*Δ mutant library was established, each mutant was passaged repeatedly on solid plates to screen for genes that might affect Type I survivor formation. Most of the mutant cells underwent senescence but a small percentage of cells were able to overcome crisis and became survivors [5]. Genomic DNA was extracted from each survival isolate, digested with the XhoI restriction enzyme, and analyzed by Southern blot with a TG probe to determine if the cells were Type I or Type II survivors (Figure 1A) (see Materials and Methods). In the first round of screening for genes affecting Type I survivor formation, we passaged two independent senescing colonies from each mutant on solid plates to obtain survivors. Because the emerging frequency of Type I survivors (∼90%) is much higher than that of Type II survivors (∼10%), most double mutants passaged on a solid plate, like the *tlc1*Δ single mutant, turned out to be Type I survivors [5]. However, if both of the two colonies picked from a single mutant strain had telomere structures consistent with that of Type II survivors, it was concluded that the gene missing in this Type II strain might contribute to Type I survivor generation and should be analyzed further. For each *tlc1*Δ *tlm*Δ mutant selected in this first round of rough screening, eight single colonies were passaged on solid plates in the second round of screening until survivors arose. When more than four colonies became Type II survivors, this TLM gene was subjected to a third round of screening in which fifty colonies of the *tlc1*Δ *tlm*Δ mutant were passaged again on solid plates. From these fifty colonies at least forty colonies typically generated survivors that could be examined. The emerging frequency of Type II survivors in each strain was then calculated (Table 1). Using this screening approach we identified eleven mutants in which the emerging frequencies of Type II survivors was elevated significantly (Table 1). Among these eleven genes, *RIF1* and *RIF2* deletion in telomerase-null *tlc1*Δ mutant generated Type II frequencies of 52.2% and 85.7% respectively (Table 1, the column of “Deleting *TLC1* in *tlm* mutants”), percentages which are consistent with a prior study performed by Teng et al. [22]. The other nine genes that affected survivor formation have never before been reported to have such a function. The Type II emerging frequencies in these nine mutants ranged from 45.7% to 93.6% (Table 1, the column of “Deleting *TLC1* in *tlm* mutants”) and were significantly elevated compared to that of the *tlc1*Δ cells, which had a Type II emerging frequency of 4% (Figure 1B). In contrast with the eleven genes that affected Type I survivor generation, the *PIF1*, helicase gene [35], [36], appeared to be essential for Type I survivor generation (discussed later).

**Table 1:** List of *S. cerevisiae* TLM genes affecting Type I versus II survivor ratio in *tlc1*Δ cells.

Gene	Tel Length	Type II Frequency		Function(Annotation from *Saccharomyces cerevisiae* Genome Database)
		Deleting *TLC1* in *tlm* mutants	Spores from tetrad dissection	
**Telomere capping or maintenance**				
*RIF1*	L	52.2% (24/46)^a^	50% (24/48)	Telomeric protein, binds to Rap1
*RIF2*	L	85.7% (42/49)	85% (34/40)	Telomeric protein, binds to Rap1
**Chromatin remodeling or modification**				
*SAP30*	S	74% (37/50)	44.7% (21/47)	Subunit of Rpd3 histone deacetylase complex
*IES3*	L	85.4% (41/48)	ND^b^	Subunit of INO80 chromatin remodeling complex
*INO80*	S	ND^c^	72% (36/50)	Subunit of INO80 chromatin remodeling complex
**DNA-dependent Transcription**				
*RPA14*	L	73.5% (36/49)	59.6% (28/47)	RNA polymerase I subunit A14
*RPB9*	S	64.6% (31/48)	90.5% (38/42)	RNA polymerase II subunit B12.6
*SOH1*	S	48% (24/50)	46.3% (19/41)	Subunit of the RNA pol II mediator complex
**rRNA processing**				
*RRP8*	L	93.6% (44/47)	56% (28/50)	Methyltransferase, pre-rRNA cleavage at site A2
**Structural constituent of ribosome**				
*RPS16B*	L	45.7% (21/46)	53.2% (25/47)	Component of the small (40S) ribosomal subunit
**Transport & membrane**				
*GUP1*	S	62.5% (30/48)	51.1% (23/45)	Plasma membrane protein in glycerol uptake

a Numbers in parenthesis shown as (a/b): “a” stands for the number of colonies which turned to be Type II survivors among 50 colonies; “b” stands for the number of post-senescence colonies which became survivors among 50 colonies.

b Spores were not generated in isogenic *tlc1*Δ *ies3*Δ double mutant.

c This *ino80*Δ strain stored in library is isogenic diploid strain. It is not feasible to delete *TLC1* in haploid *ino80*Δ mutant having severe growth defect. We initiated our experiments from dissected *tlc1*Δ *ino80*Δ spores.

Very recently, Chang et al. showed that the long telomeres in *rif1*Δ *tlc1*Δ and *rif2*Δ *tlc1*Δ mutants were preferentially extended by a recombination pathway and senescent cells with long telomeres were more efficient at bypassing senescence via the Type II survivor pathway [37]. These led Chang et al. to propose that *rif1*Δ *tlc1*Δ and *rif2*Δ *tlc1*Δ mutants affect the ratio of survivor types by altering telomere length at the point of senescence [37]. In order to examine the idea that telomere length affects the type of survivor generated, we generated eleven *TLC1*/*tlc1*Δ *TLM*/*tlm*Δ diploid strains and performed tetrad dissections to obtain *tlc1*Δ single and *tlc1*Δ *tlm*Δ double mutants (Table 1, the column of “Spore from tetrad dissection”). Because the *ino80*Δ *tlc1*Δ double mutant used in the previous experiments was obtained from tetrad dissection, it was not included in this experiment. Fifty senescing clones of the other ten mutant strains, including *tlc1*Δ single mutants from each diploid mutant, were streaked on plates until survivors arose. Telomere structures of the survivors generated on plates were examined by Southern blot (Figure S1). A representative Southern blot result of *rpa14*Δ *tlc1*Δ mutant is shown in Figure 1C. The results of these experiments are summarized below and are listed in the column of “Spore from tetrad dissection” in Table 1. The frequency of Type II survivor formation in the *sap30*Δ *tlc1*Δ, *rpa14*Δ*tlc1*Δ, *rrp8*Δ *tlc1*Δ and *gup1*Δ *tlc1*Δ double mutants was decreased when compared to that of the corresponding double mutant that had not been through sporogenesis. The frequency of Type II survivor formation in the *rpb9*Δ *tlc1*Δ or *rps16b*Δ *tlc1*Δ double mutants was increased when compared to that of the corresponding double mutant that had not been through sporogenesis. The frequency of Type II survivor formation in *rif1*Δ *tlc1*Δ, *rif2*Δ *tlc1*Δ and *soh1*Δ *tlc1*Δ double mutants did not change significantly. Recovery of the *ies3*Δ *tlc1*Δ double mutant from sporogenesis was not successful. We also examined telomere length around the time of survivor formation and found that similar to the *rif1*Δ *tlc1*Δ and *rif2*Δ *tlc1*Δ mutants, the critical telomere length in *gup1*Δ *tlc1*Δ and *ino80*Δ *tlc1*Δ mutants was about 50 bp longer than those in *tlc1*Δ single mutants from the same crosses (Figure S2). However, in *soh1*Δ *tlc1*Δ and *rpb9*Δ *tlc1*Δ mutants, the critical telomere lengths were about 30 bp shorter than those in *tlc1*Δ mutants from the same crosses (Figure S2). Additionally, in the *rps16b*Δ *tlc1*Δ, *sap30*Δ *tlc1*Δ and *rrp8*Δ *tlc1*Δ mutants, the critical telomere lengths were slightly longer (<30 bp) than those in *tlc1*Δ mutants from the same crosses (Figure S2). In the *rpa14*Δ *tlc1*Δ mutant, the critical telomere length was similar to that in *tlc1*Δ mutant from the same cross (Figure S2). Our data support the idea put forth by Cheng et al. that telomere length affects survivor formation [37]. Our data also show the frequency of Type II emergence in the nine mutants we identified ranged from 44.7% to 90.5%, which was much higher than the Type II emerging frequencies of less than 10% that were usually observed in *tlc1*Δ cells (Table 1 and Figure S1) [5].

### INO80 chromatin remodeling complex affects the emerging frequency of Type I survivor generation

**Figure 2:**
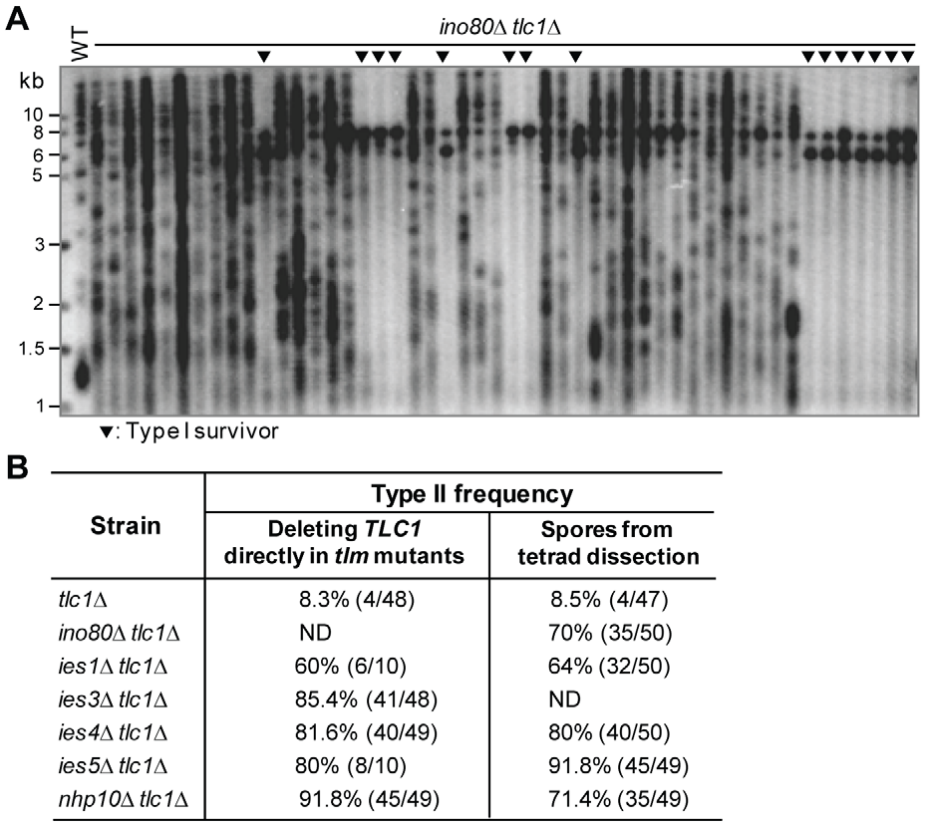
The effect of the Ino80 complex on survivor formation. (A) Fifty independent survivor colonies of the *ino80*Δ *tlc1*Δ mutant, which was generated from INO80/*ino80*Δ *TLC1*/*tlc1*Δ diploid mutant, were randomly picked and their genomic DNA was isolated for Southern blot assay using a TG_1–3_ probe. The black triangles indicate Type I survivors. (B) Chart of Type II survivor frequencies in the mutants of Ino80 complex subunits. ND: not done (see Table 1).

The INO80 complex is one of the ATP-dependent chromatin remodeling complexes that can move or evict nucleosomes, thereby changing chromatin structure and affecting the accessibility of DNA to other factors [38]. The yeast INO80 complex contains multiple subunits, including five essential and ten (Ino80, Ies1, Ies2, Ies3, Ies4, Ies5, Ies6, Taf14, Arp8 and Nhp10) non-essential subunits [38]. A recent study has shown that Ies3 interacts with the telomerase component Est1 [34]. In *est1*Δ cells, deleting *IES3* or *ARP8* caused a delay of survivor generation in liquid culturing [34], suggesting that the INO80 complex affects telomere recombination. In our survivor screening we noted that two subunits in the INO80 complex, Ino80 and Ies3, significantly affected the generation of Type I survivors (Table 1). When passaged on solid medium, the *ino80*Δ *tlc1*Δ and *ies3*Δ *tlc1*Δ mutants produced Type II survivors at frequencies of 70% and 85.4% respectively (Figure 2A and 2B), which were significantly elevated in comparison with the 8.3% we observed in *tlc1*Δ cells (Figure 2B and Figure S3A). These results suggested that the INO80 complex may be required for efficient Type I survivor formation. To examine this possibility further we examined the impact of depleting each of the other four non-essential subunits of the INO80 complex on the efficiency of Type I survivor formation in *tlc1*Δ cells. The Southern blot results revealed that the deletion of each of the non-essential INO80 subunits *IES1*, *IES4*, *IES5* and *NHP10* led to the generation of more Type II than Type I survivors (Figure S3). The frequency of Type II emergence in each of these mutants in *tlc1*Δ cells was above 60% (Figure 2B), which was much higher than that of the *tlc1*Δ single mutant. These results indicate that the INO80 complex greatly influences the emerging ratio of Type I vs Type II survivors.

### Pif1 is required for Type I recombination

*PIF1* is a non-essential gene which encodes a 5′ to 3′ DNA and DNA/RNA helicase in *S. cerevisiae* [36], [39]. Previous studies have demonstrated that Pif1 can be translated from different start sites and has two forms which are localized to either the mitochondria or the nucleus [35], [40]. In the mitochondria Pif1 affects recombination of mitochondrial DNA (mtDNA) and plays an important role in maintaining mtDNA stability [41]–[43]. In the nucleus, Pif1 inhibits telomere lengthening by removing telomerase from telomeric DNA [35], [44] and participates in Okazaki fragment maturation [45], [46] and ribosomal DNA replication [47]. Additionally, Pif1 is able to unwind G-quadruplex structures *in vitro* [48], and likely acts on these structures *in vivo* as well [48], [49].

**Figure 3:**
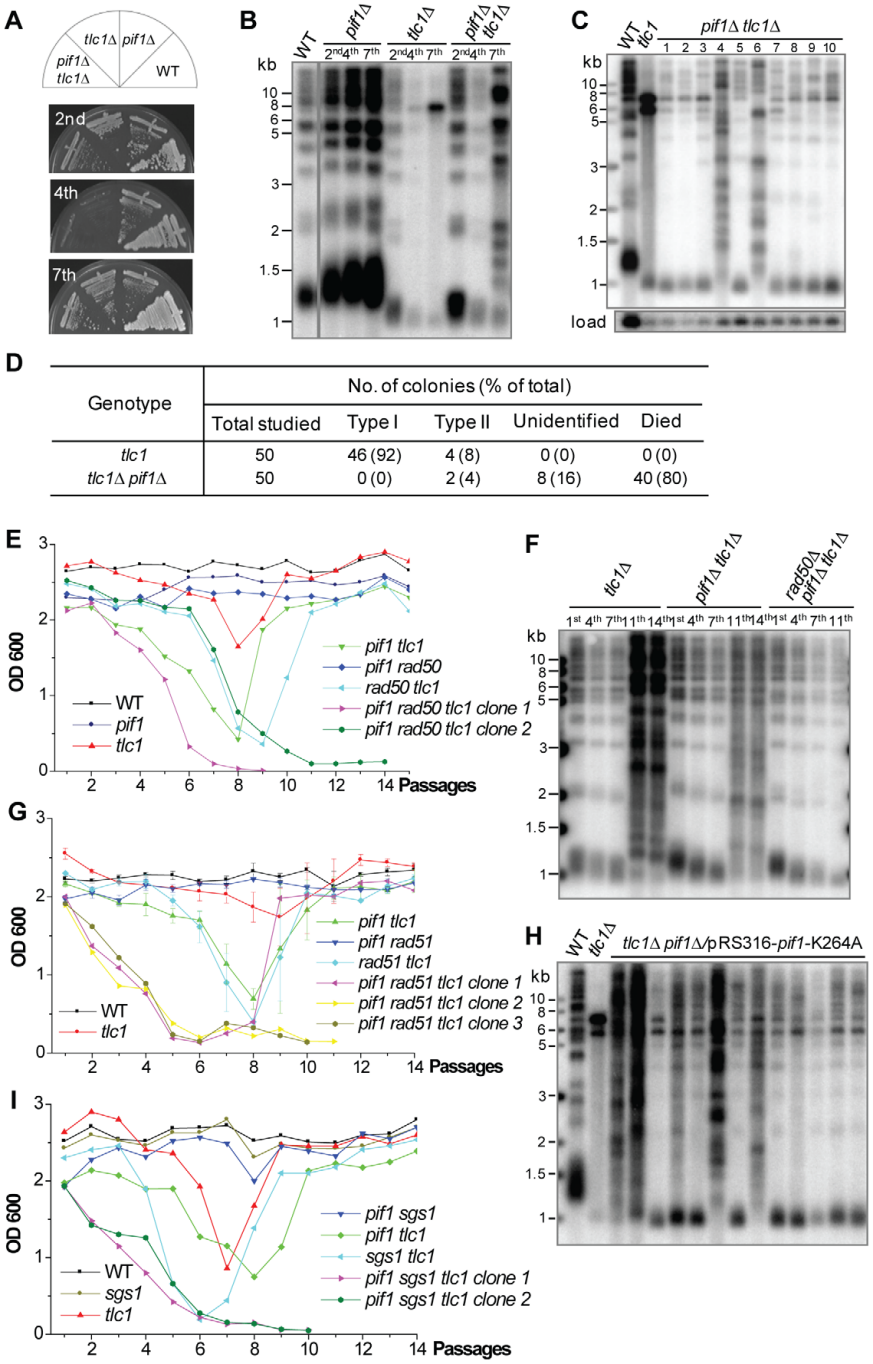
Type I recombination requires Pif1 helicase. (A) The heterozygous diploid mutant, in which one copy of *TLC1* and *PIF1* were deleted, was sporulated and dissected, and then individual spores from tetrads were restreaked seven times to allow survivors to form. (B) The genomic DNA of independent colonies from each mutant assayed in (A) was subjected to Southern blot analysis using a TG probe after the 2^nd^, 4^th^ and 7^th^ streaking as indicated. (C) Fifty independent *tlc1Δ pif1Δ* senescing colonies were picked and restreaked to generate survivors and the genomic DNA of ten living post-senescence colonies at the 7^th^ streaking was subjected to Southern blot analysis. The blot membrane was re-probed with a *CDC15* probe as a loading control [50]. (D) Statistical results of the survivors generated from fifty independent colonies in *tlc1Δ* and *pif1Δ tlc1Δ* mutants. (E) The heterozygous diploid triple mutant of *PIF1*/*pif1*Δ *RAD50*/*rad50*Δ *TLC1*/*tlc1*Δ was dissected and the isogenic spores were subjected to cell viability assay in liquid culture. The results of two spores for *pif1*Δ *tlc1*Δ *rad50*Δ mutant are shown. (F) Genomic DNA of the *tlc1*Δ, *pif1*Δ *tlc1*Δ and *pif1*Δ *rad50*Δ *tlc1*Δ strains assayed in (E) was subjected to a Southern blot analysis after the 1^st^, 4^th^, 7^th^, 11^th^ and 14^th^ passages. (G) The heterozygous diploid triple mutant of *PIF1*/*pif1*Δ *RAD51*/*rad51*Δ *TLC1*/*tlc1*Δ was dissected and the isogenic spores were subjected to a liquid culture cell viability assay. (H) Fifty independent *tlc1Δ pif1Δ*/pRS316-*pif1*-K264A senescing colonies were serially restreaked and DNA of thirteen surviving post-senescence colonies after the 7^th^ streaking was subjected to the Southern blot assay shown. (I) The heterozygous diploid triple mutant of *PIF1*/*pif1*Δ *SGS1*/*sgs1*Δ *TLC1*/*tlc1*Δ was dissected and the isogenic spores were subjected to cell viability assay in liquid culture. The results of two spores for *pif1*Δ *tlc1*Δ *sgs1*Δ mutant are shown.

In our primary screening the *pif1*Δ *tlc1*Δ double mutant had difficulties generating survivors on solid medium, and as a result most clones died out during sequential streaks. The *pif1*Δ *tlc1*Δ clones that overcame senescence on solid medium showed a Type II survivor pattern (Figure 3A and 3B), suggesting that Pif1 promotes Type I survivor formation. To further validate the role of Pif1 in Type I survivor generation, we streaked fifty independent *pif1Δ tlc1Δ* colonies on plates. We noted that forty post-senescence colonies (80%) died during the sequential streaks, indicating that deletion of *PIF1* in telomerase-null strains inhibits the creation of post-senescence survivors. The other 10 colonies also underwent senescence, but were able to generate survivors at the 7th streaking. Cells at this stage were harvested, and their telomeres were examined by Southern blot assay (Figure 3C). Only two colonies (4%), which grew at a normal rate, gave rise to type II survivors (Figure 3C and 3D), indicating that type II survivors can indeed form in the absence of Pif1. Interestingly, eight colonies (16%) of extremely slow growing survivors showed distinct patterns of telomeric DNA without either long heterogeneous TG tracts or substantial Y' amplification (Figure 3C and 3D), suggesting that a new type of survivor emerged in *pif1Δ tlc1Δ* post-senescence cells. In these cells the terminal TG tracts seemed to be even shorter than that in Type I survivors but were unexpectedly maintained during subsequent passages. This abnormality of telomeric DNA was also observed by Dewar et al. [50]. Nevertheless our results suggested that Pif1 is required for Type I survivor formation. To confirm this further, since *RAD50* and *RAD51* are respectively required for Type II and Type I survivor formation we checked whether survivors could form in either a *rad50*Δ *pif1*Δ *tlc1*Δ or a *rad51*Δ *pif1*Δ *tlc1*Δ triple mutant. The isogenic *rad50*Δ *pif1*Δ *tlc1*Δ or *rad51*Δ *pif1*Δ *tlc1*Δ spores were dissected and serially passaged in liquid culture. As expected, two spores of the *rad50*Δ *pif1*Δ *tlc1*Δ triple mutant underwent senescence gradually and virtually died out at the 9^th^ or 11^th^ passage (Figure 3E). A Southern blot analysis showed that *tlc1*Δ and *pif1*Δ *tlc1*Δ mutants displayed Type II survivor telomere structures after eleven passages, whereas *rad50*Δ *pif1*Δ *tlc1*Δ mutant did not (Figure 3F). These results further support our claim that Pif1 is required for Type I survivor generation. For the *rad51*Δ *pif1*Δ *tlc1*Δ triple mutant, three spores behaved differently in liquid culture. One spore could generate survivors, while the other two spores could not (Figure 3G), suggesting that Pif1 might also affect Type II survivor generation.

To investigate whether Pif1's helicase activity is required for Type I survivor formation, we constructed the pRS316-*pif1*-K264A plasmid and transformed it into *pif1*Δ cells, as the lysine residue of 264 in the ATP-binding domain of Pif1 is essential for Pif1's helicase activity [35]. Fifty senescing colonies of *pif1*Δ *tlc1*Δ/pRS316-*pif1*-K264A strain were randomly selected and passaged to allow survivors to generate. Thirty-seven post-senescence colonies died during the sequential streaks, while thirteen colonies generated survivors. A Southern blot analysis revealed that the telomere structures of these post-senescence survivors were very similar to those of the *pif1*Δ *tlc1*Δ mutant survivors (Figure 3H). We therefore concluded that Pif1's helicase activity plays a key role in telomeric DNA recombination.

Helicases are nucleic acid-dependent ATP-ases that are capable of unwinding DNA or RNA duplex substrates and play important roles in almost every cellular process including DNA replication and repair, transcription, translation, RNA processing and so on [51], [52]. In *S. cerevisiae*, there are 132 open-reading-frames that encode helicase or helicase-like proteins [35]. Thirteen of them have been shown to have DNA helicase activity. We knocked out *TLC1* in each of these thirteen DNA helicase gene mutants (Figure S4) and carried out survivor screenings to investigate if these genes affect Type I or Type II survivor generation. In contrast with *PIF1*, the other twelve DNA helicase genes and *TLC1* double deletion mutants generated Type I survivors on solid medium, indicating that they are not essential for Type I survivor formation (Figure S4A). In liquid medium, *sgs1*Δ *tlc1*Δ cells generated Type I survivors, while the other twelve DNA helicase genes and *TLC1* double deletion mutants generated Type II survivors after passaging 12 times (about 200 population doublings) (Figure S4B). This result is consistent with a previous report which shows Sgs1 helicase is required for Type II survivor formation [53]. We obtained the *pif1*Δ *sgs1*Δ *tlc1*Δ triple mutant dissected from the heterozygous *PIF1*/*pif1*Δ *SGS1*/*sgs1*Δ *TLC1*/*tlc1*Δ diploid mutant. The *pif1*Δ *sgs1*Δ *tlc1*Δ mutant was cultured in liquid medium and no survivors were recovered (Figure 3I). It was therefore concluded that Pif1 and Sgs1 may define the Type I and Type II survivor formation pathways respectively.

### Screening of TLM gene deletion library in liquid medium identifies genes affecting Type II survivor formation

**Figure 4:**
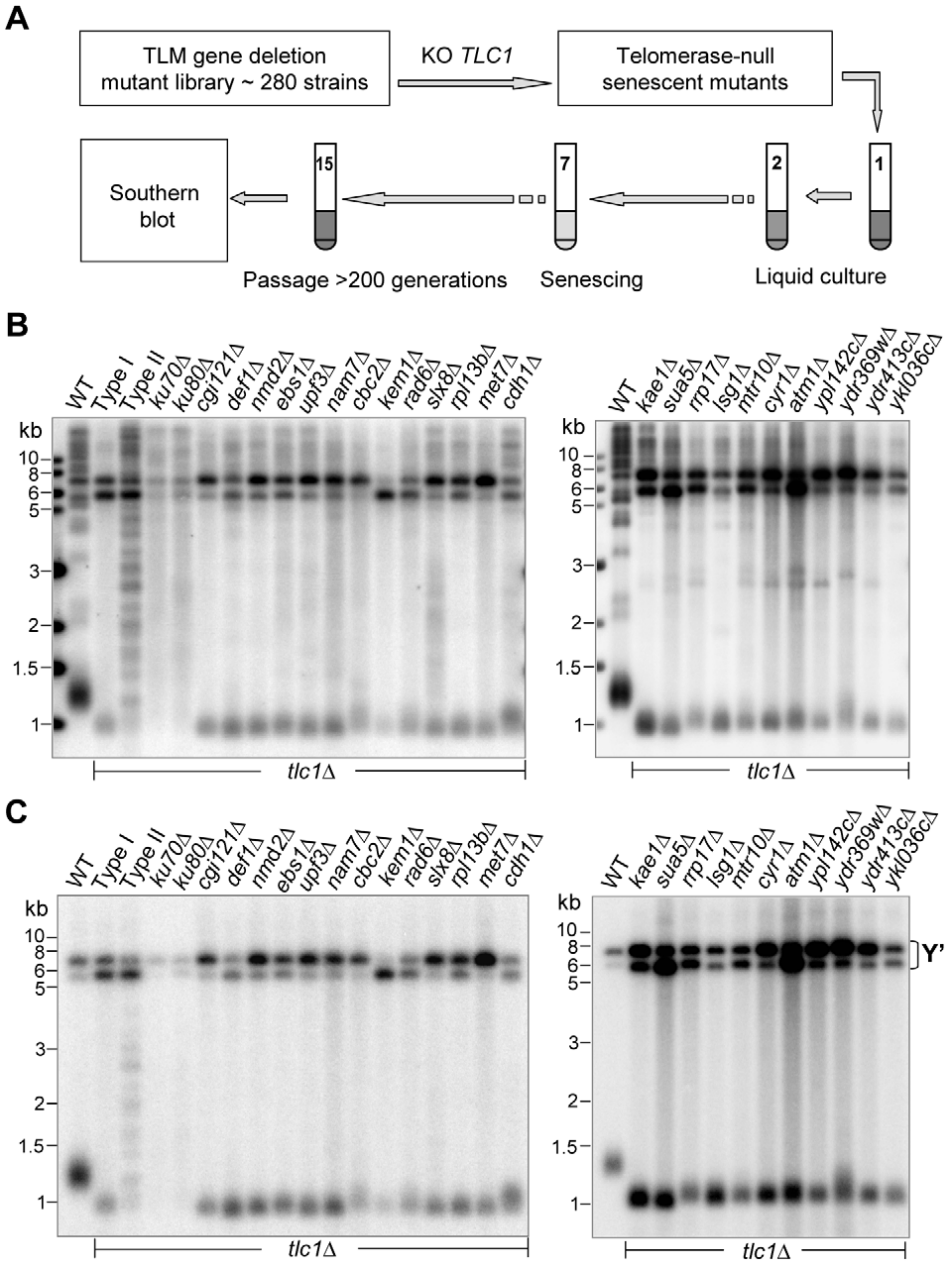
Southern blot analyses of Type I survivors generated in *tlm*Δ *tlc1*Δ mutants in liquid culture. (A) Schematic illustration of the screening procedures for genes that affect Type II survivor formation (refer to details in main text). (B and C) Southern blot analyses of survivor types in the *tlc1*Δ strain (Type I and Type II serves as controls) and twenty-six *tlm*Δ *tlc1*Δ double mutants using a TG probe (B) and a Y' probe (C).

In order to screen for genes that might affect Type II survivor formation, we grew the 280 telomerase-null *tlm*Δ mutants serially in liquid medium to generate survivors (Figure 4A) [5]. If Type II survivors arise, they eventually out-compete their Type I counterparts in liquid culture because of their aforementioned growth advantage [5]. There were, however, some strains that lacked the genes required for Type II survivor formation, and thus generated only Type I survivors. The viability of these senescing mutants was recorded during passages and survivor cells were harvested at the end of serial culturing. The genomic DNA of the liquid-cultured cells was isolated and subjected to Southern blot with a telomeric TG_1–3_ probe. Twenty-four *tlc1*Δ *tlm*Δ double mutants formed Type I survivors, suggesting these twenty-four genes were required for Type II survivor formation (Figure 4B and Table 2). To further confirm the Type I phenotypes of these mutants, we used a Y' probe and performed Southern blot hybridization to examine the DNA structure. The results clearly showed significant amplification of Y'-elements, a characteristic typical of Type I survivors (Figure 4C).

**Table 2:** List of *S. cerevisiae* TLM genes required for Type II survivor formation.

Gene	Tel Length	Function (Annotation from *Saccharomyces cerevisiae* Genome Database)
**Telomere capping or maintenance**		
*CGI121*	S	KEOPS complex
*KAE1*	S	KEOPS complex
*SUA5*	S	Telomeric ssDNA-binding, t6A modification (tRNA)
*DEF1*	S	Interacted with Rrm3p, RNAPII degradation factor
**Nonsense-mediated decay**		
*NMD2*	S	Nonsense-mediated decay
*UPF3*	S	Nonsense-mediated decay
*EBS1*	S	NMD; inhibition of translation; EST1 homologue
*NAM7*	S	NMD; ATP-dependent RNA helicase
*CBC2*	L	Component of the spliceosomal commitment complex
**Protein ubiquitination**		
*RAD6*	S	E2 ubiquitin-conjugating enzyme in DNA repair
*SLX8*	L	Slx5-Slx8 substrate-specific ubiquitin ligase complex
**rRNA processing**		
*XRN1*	S	Conserved 5′-3′ exonuclease component in mRNA decay
*RRP17*	S	Exonuclease for 5′ end processing of pre-60S ribosomal RNA
**Structural constituent of ribosome**		
*RPL13B*	S	Component of the large (60S) ribosomal subunit
**Cell cycle**		
*CDH1*	L	Cell-cycle regulated activator of APC
**Transport & membrane**		
*MET7*	S	Folylpolyglutamate synthetase
*ATM1*	S	Mitochondrial inner membrane ATP-binding cassette transporter
*CYR1*	L	Adenylate cyclase
*MTR10*	S	Nuclear import receptor
*LSG1*	S	Putative GTPase; required for Nmd3p release from 60S subunits
**Unknown**		
*YPL142C*	S	Dubious ORF
*YDR396W*	S	Dubious ORF
*YDR413C*	S	Dubious ORF
*YGL069C*	S	Dubious ORF

Among these twenty-four genes, twenty-two had never before been identified for their involvement in Type II survivor formation (Table 2). The two genes identified in our screening that have been previously reported to maintain such a function include *SUA5* and *DEF1* [16], [18]. It is important to note that survivors generated in *tlc1*Δ *yku70*Δ or *tlc1*Δ *yku80*Δ cells exhibited distinctive telomeric DNA patterns that differed from classical Type I and Type II survivor structures (Figure 4B and 4C, left panels) [54], [55]. Moreover, both *tlc1*Δ *yku70*Δ and *tlc1*Δ *yku80*Δ cells exhibited more rapid senescence and became survivors as soon as the telomeric DNA from germinating spores could be examined, observations which are consistent with earlier reports [55], [56]. The results of the *yku70* and *yku80* mutants were presented in this section with the other mutants which displayed Type I survivors because survivor generation in *tlc1*Δ *yku70*Δ and *tlc1*Δ *yku80*Δ mutants is more dependent upon *RAD51* than *RAD50* [54].

### Type II survivor formation involves the Rad6-Bre1 pathway

**Figure 5:**
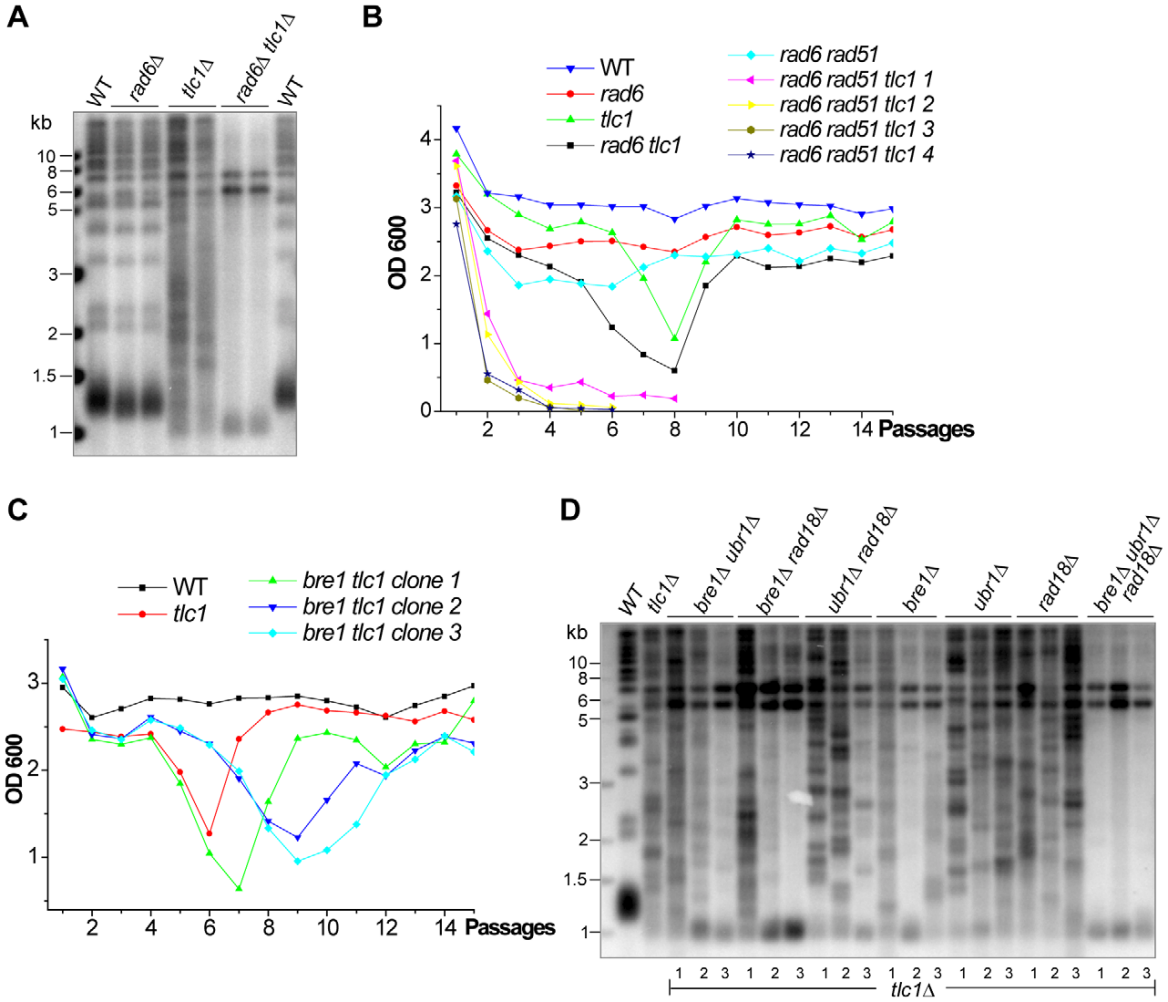
The effect of the Rad6 on survivor formation. (A) The isogenic strains of *tlc1*Δ, *rad6*Δ and *rad6*Δ *tlc1*Δ were serially passaged in liquid medium to generate survivors and then genomic DNA of survivors was subjected to Southern blot analysis. (B) The heterozygous diploid *RAD6*/*rad6*Δ *RAD51*/*rad51*Δ *TLC1*/*tlc1*Δ strain was sporulated and tetrads were dissected, and the spores with the indicated genotypes (including four clones of *rad6*Δ *rad51*Δ *tlc1*Δ were subjected to a cell viability assay. (C) The heterozygous diploid *TLC1*/*tlc1*Δ *RAD18*/*rad18*Δ *BRE1*/*bre1*Δ *UBR1*/*ubr1*Δ mutant was sporulated and tetrads were dissected, and the spores with the indicated genotypes were subjected to a cell viability assay. Cell viabilities of three clones of the *bre1*Δ *tlc1*Δ mutant are shown in this panel, while those of the *ubr1*Δ *tlc1*Δ, *rad18*Δ *tlc1*Δ, *bre1*Δ *ubr1*Δ *tlc1*Δ, *bre1*Δ *rad18*Δ *tlc1*Δ, *ubr1*Δ *rad18*Δ *tlc1*Δ, and *rad18*Δ *bre1*Δ *ubr1*Δ *tlc1*Δ mutants are shown in Figure S5. (D) Genomic DNA of the isogenic strains (indicated on top of the panel) was isolated and subjected to Southern blot assay. Three colonies (labeled at the bottom of the panel) of each strain were examined.

As mentioned above, we identified twenty-two genes not previously known to be required for Type II survivor formation (Table 2). *RAD6* remains of particular interest as previous studies have shown that *RAD6* plays important roles in recombinational repair [57]. Rad6 is an E2 ubiquitin-conjugating enzyme and it interacts with three E3 ubiquitin ligases (Bre1, Rad18 and Ubr1) known to be involved in different DNA repair pathways [58], [59]. Rad6 and Bre1 are responsible for H2B-K123 ubiquitination, which is required for H3-K4 methylation [60]. Rad6 and Rad18 are involved in post-replication repair via their role in ubiquitination of PCNA [61]. Rad6 and Ubr1 have been linked to DNA repair through their function in degradation of cohesin [62]. Our Southern blot analysis showed that *rad6*Δ *tlc1*Δ double mutant cells in liquid culture generated only Type I survivors (Figure 5A), suggesting that Rad6 is required for Type II survivor formation. To validate this result, we knocked out *RAD51*, which is required for Type I survivor formation, in the *rad6*Δ *tlc1*Δ cells. All four clones of the *rad6*Δ *rad51*Δ *tlc1*Δ mutant underwent senescence and were unable to generate survivors (Figure 5B), confirming that Rad6 is required for Type II survivor formation.

In order to determine the downstream pathways utilized by Rad6 during Type II survivor generation we constructed the heterozygous diploid strain of *TLC1*/*tlc1*Δ *RAD18*/*rad18*Δ *BRE1*/*bre1*Δ *UBR1*/*ubr1*Δ. The isogenic haploid *tlc1*Δ strains of single-, double-, triple- and quadruple-mutants were derived by sporulation. Three independent colonies of *tlc1*Δ *bre1*Δ, *tlc1*Δ *ubr1*Δ, *tlc1*Δ *rad18*Δ, *tlc1*Δ *bre1*Δ *ubr1*Δ, *tlc1*Δ *bre1*Δ *rad18*Δ, *tlc1*Δ *ubr1*Δ *rad18*Δ, and *tlc1*Δ *rad18*Δ *bre1*Δ *ubr1*Δ were passaged in liquid medium to allow survivor formation. The analysis of strain viability is shown in Figure 5C and Figure S5. An aliquot of each liquid-grown survivor was harvested on the second day of recovery, and its telomeric DNA was examined by Southern blot (Figure 5D). The *tlc1*Δ *ubr1*Δ, *tlc1*Δ *rad18*Δ and *tlc1*Δ *ubr1*Δ *rad18*Δ survivors displayed no obvious amplification of Y'-subtelomeric elements whereas the *tlc1*Δ *bre1*Δ, *tlc1*Δ *bre1*Δ *ubr1*Δ and *Δtlc1*Δ *bre1*Δ *rad18*Δ survivors that lacked the *BRE1* gene displayed significant Y'-element amplification (Figure 5D). These data suggest Bre1 plays an even more positive regulatory role in Type II survivor generation than Ubr1 and Rad18. Interestingly, the *tlc1*Δ *rad18*Δ *bre1*Δ *ubr1*Δ mutant cells only allowed the development of Type I survivors. These results indicate that Rad6 functions through its downstream pathways and most importantly Bre1 to promote Type II survivor formation.

### The KEOPS complex is required for Type II recombination

**Figure 6:**
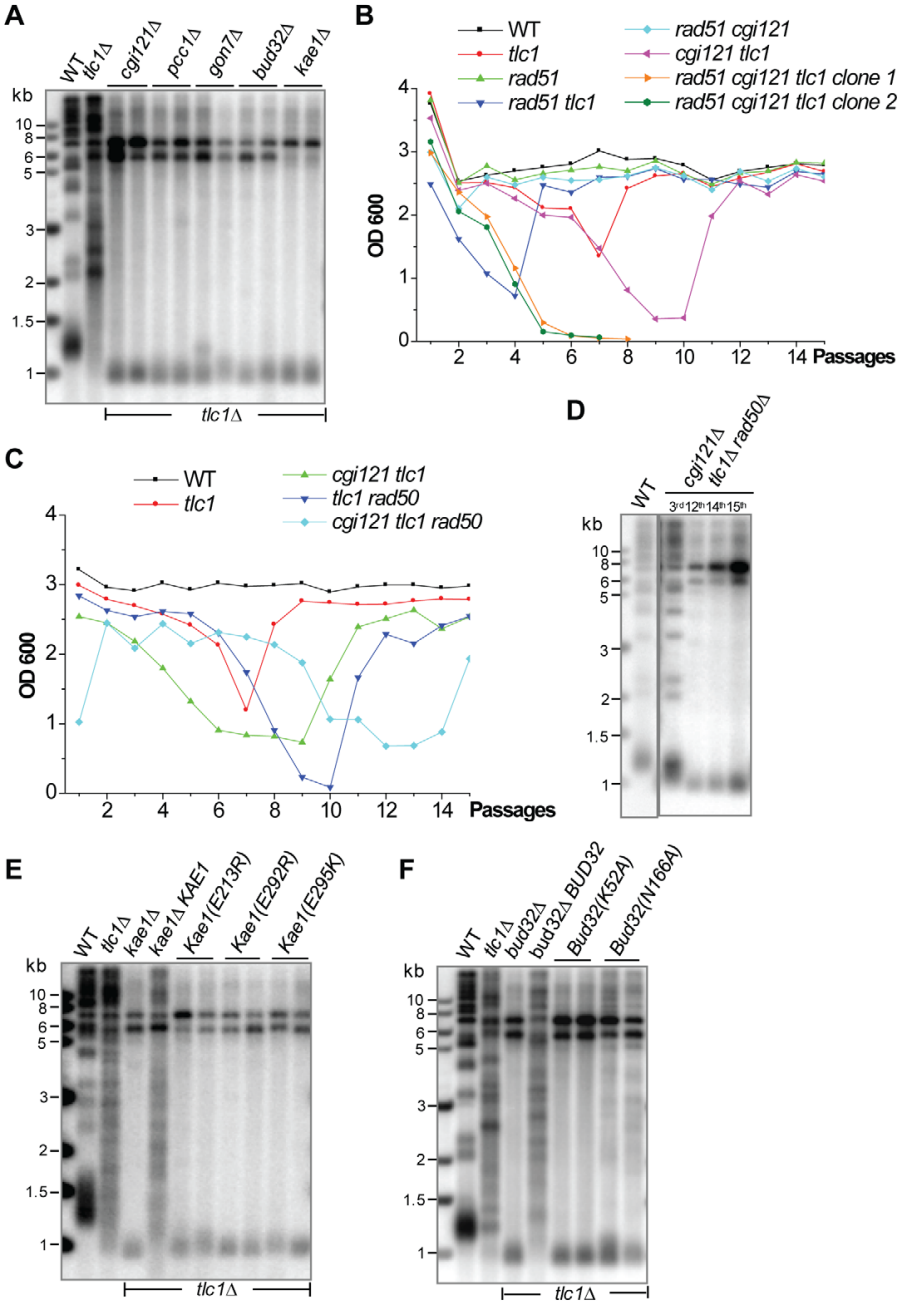
Each subunit of the KEOPS complex is essential for Type II recombination. (A) Southern blot of the genomic DNA of the survivors generated by serial liquid culturing of *cgi121*Δ *tlc1*Δ, *pcc1*Δ *tlc1*Δ, *gon7*Δ *tlc1*Δ, *bud32*Δ *tlc1*Δ and *kae1*Δ *tlc1*Δ mutants. (B) Liquid culture cell viability analyses of sibling spores generated in the diploid CGI121/*cgi121*Δ *TLC1*Δ*tlc1*Δ *RAD51/rad51*Δ strain. The *cgi121*Δ *tlc1*Δ *rad51*Δ triple mutant died out at the 7^th^ passage. (C) Cell viability analysis of sibling spores generated in the diploid *CGI121*/*cgi121*Δ *TLC1*Δ*tlc1*Δ *RAD50*/*rad50*Δ strain. Two spores were analyzed in parallel and the results were similar. (D) Southern blot analysis of telomere DNA in survivors generated by serial liquid culturing of the *cgi121*Δ *tlc1*Δ *rad50*Δ triple mutant. (E) and (F) Southern blot analyses of telomere DNA in survivors generated by serial liquid culturing of the *tlc1*Δ *kae1*(E213R), *tlc1*Δ *bud32*(E292R), *tlc1*Δ *bud32*(E295K) mutants (in E), *tlc1*Δ *bud32*(K52A) and *tlc1*Δ *bud32*(N166A) mutants (in F).

In addition to *RAD6*, *CGI121* and *KAE1* were also identified during our liquid-culture screen as contributing to Type II survivor formation (Table 2). Cgi121 and Kae1 belong to the KEOPS complex, which is evolutionarily conserved from archaea to mammals [63]. In *S. cerevisiae*, the KEOPS complex consists of five subunits (Cgi121, Bud32, Kae1, Gon7 and Pcc1) and plays multiple roles in transcription, tRNA modification (t6A), chromosome segregation and telomere uncapping-elongation [29], [64]–[66]. The deletion mutants of *BUD32* and *GON7* were in our original TLM library but in our initial screening the severe growth defects of the *bud32*Δ and *gon7*Δ haploid strain made it impossible for us to knock-out *TLC1*. *PCC1* was not in the 280 TLM gene list, and therefore was not covered in our initial screening. In order to determine whether Bud32, Gon7 and Pcc1 were also involved in telomere recombination, we constructed the heterozygous diploid mutants in which one copy of *TLC1* and *BUD32*, *GON7* or *PCC1* were deleted. The double mutants of *bud32*Δ *tlc1*Δ, *gon7*Δ *tlc1*Δ and *pcc1*Δ *tlc1*Δ were obtained from tetrad dissection and were serially passaged in liquid medium. All the survivors displayed Type I patterns of Y' amplification (Figure 6A), indicating that Type II recombination could not take place in the absence of Bud32, Gon7 or Pcc1.

To further confirm the critical role the KEOPS complex plays in telomere recombination, we tested whether survivor formation in *cgi121*Δ *tlc1*Δ cells would be affected in the absence of *RAD51* or *RAD50*. Unfortunately, we could not examine the genetic interaction between *RAD51* or *RAD50* and the other four KEOPS subunits in telomere recombination because the *bud32*Δ, *kae1*Δ, *gon7*Δ and *pcc1*Δ mutants all exhibited severe growth defects. Therefore we focused on *CGI121* by generating a heterozygous diploid strain in which one copy of *TLC1*, *CGI121* and *RAD51* (or *RAD50*) was deleted. The isogenic strains of single, double and triple mutants were derived from tetrad dissection and serially cultured in liquid medium. The *cgi121*Δ *tlc1*Δ *rad51*Δ triple mutant died out rapidly, while other *tlc1*Δ mutants were able to recover robust growth when survivors arose (Figure 6B). Consistently, the *cgi121*Δ *tlc1*Δ *rad50*Δ triple mutant was able to bypass the senescence crisis by generating Type I survivors (Figure 6C and 6D). These results support the conclusion that *CGI121* and likely the entire KEOPS complex is required for Type II recombination.

Previous studies have shown that Kae1 has ATP-binding activity and Bud32 acts as a protein kinase and the activities of both of these gene products appear to be essential for all the roles played by the KEOPS complex [29], [63], [66], [67]. Based on the previous biochemical and structural analyses of Kae1 and Bud32 [63], we constructed *kae1*(E213R) *tlc1*Δ, *bud32*(K52A) *tlc1*Δ and *bud32*(N166A) *tlc1*Δ double mutant strains in which the Kae1 ATP-binding site and the Bud32 kinase catalytic sites were mutated. These mutants were unable to generate Type II survivors, but rather exclusively developed Type I survivors when cultured in liquid medium (Figure 6E and 6F). Likewise, the *kae1*(E292R) and *kae1*(E295K) mutants, which no longer maintain an interation between Kae1 and Bud32, also displayed a defect in Type II survivor generation (Figure 6E). These data indicate that both the Kae1-Bud32 interaction and their biochemical activities were indispensable for Type II telomere recombination. We therefore concluded that the whole KEOPS complex was necessary for Type II recombination.

### Some TLM genes involved in telomere recombination also affect DNA recombination in general

**Figure 7:**
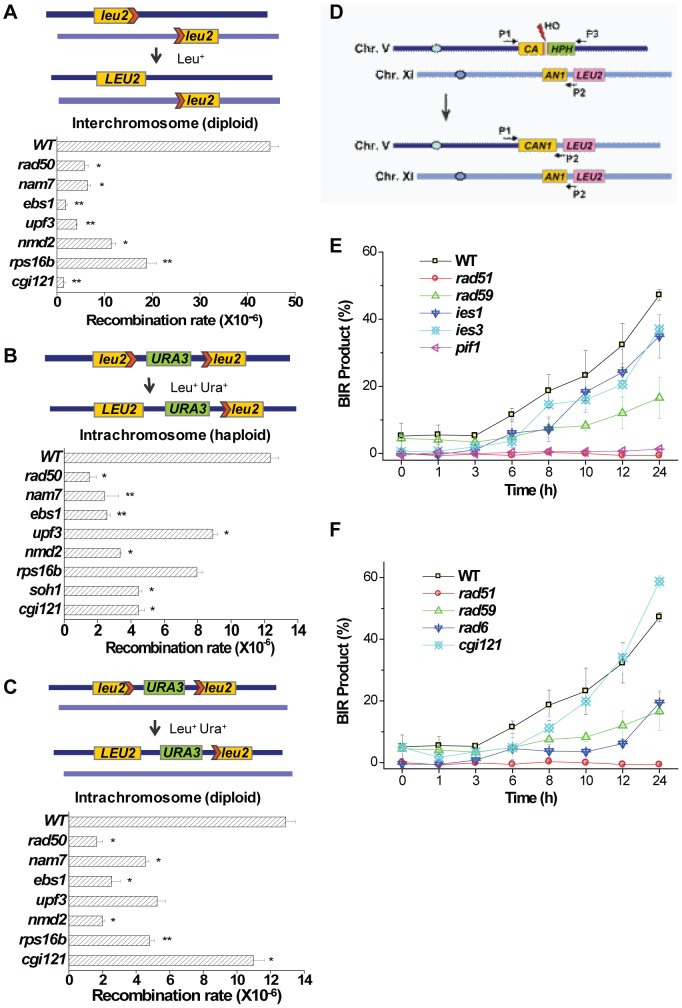
General homologous recombination activities in *nam7*Δ, *ebs1*Δ, *upf3*Δ, *nmd2*Δ, *rps16b*Δ, *soh1*Δ, and *cgi121*Δ mutants, and break-induced-replication efficiencies in *ies1*Δ, *ies3*Δ, *pif1*Δ, *rad6*Δ, and *cgi121*Δ mutants. (A) Inter-chromosomal recombination assay in the indicated homozygous diploid deletion mutants. (B) Intra-chromosomal recombination assay in the indicated haploid mutants. (C) Intra-chromosomal recombination assay in the indicated homozygous diploid deletion mutants. The upper panels are the schematic illustrations of each recombination event. The lower panels show the measured recombination rates in the indicated mutants. These assays were performed as reported previously [72], [73] and the statistical significance was indicated as follows: **P-value<0.02 and *P-value<0.05. The *rad50*Δ mutant was included as a positive control. We failed in constructing *soh1*Δ homozygous mutants in (A) and (C) because the mating efficiency of this mutant was extremely low. (D) Schematic illustration of the system to detect break-induced-replication (BIR) efficiencies as reported in Lydeard et al [74]. After galactose induction, HO endonuclease causes a break in the indicated site, BIR repair process generates an intact *CAN1* marker which can be detected by PCR procedures using primers P1 and P2. (E and F) BIR efficiencies were measured in *pif1*Δ, *ies1*Δ and *ies3*Δ mutants (E), and *rad6*Δ and *cgi121*Δ mutants (F). Semi-quantitative PCR was used to measure BIR efficiency as shown in Figure S6.

Previously, several labs performed genome-wide screens searching for genes that affect DNA repair and/or recombination and dozens of genes were documented [68]–[71]. In this study we have identified ten genes which affect Type I telomere recombination and twenty-two genes which affect Type II telomere recombination. Fifteen of these genes have already been reported to have potential roles in general DNA repair and/or recombination (Table S2). In order to determine whether the other seventeen genes also play roles in general DNA repair/recombination, we performed three assays used previously [72], [73] to examine relative levels of inter-chromosomal homologous recombination (Figure 7A) and intra-chromosomal homologous recombination in haploid (Figure 7B) and diploid strains (Figure 7C). Each assay detected genomic gene conversion events through the recovery of an intact *LEU2* marker by the integration of two seperated fragments (Figure 7A–7C, upper panels). Ten of the seventeen mutants we tested exhibited an extremely slow growth phenotype and were not viable for testing using the general recombination assays. The remaining seven mutants (*nam7*Δ, *ebs1*Δ, *upf3*Δ, *nmd2*Δ, *rps16b*Δ, *soh1*Δ and *cgi121*Δ) showed decreased activities in inter- or intra-chromosomal homologous recombination (Figure 7A–7C). Therefore it is likely that these seven genes participate in telomere recombination as well as recombination at other genomic loci.

### The INO80 complex, Pif1, and Rad6 affect telomere recombination through break-induced-replication mechanism

Break-induced-replication (BIR) only requires one free DNA end to take place and it has been proposed to be the principal mechanism for telomere recombination and survivor generation [14]. To examine whether the INO80 complex, Pif1, Rad6 and the KEOPS complex participate in telomere recombination via Rad51-dependent BIR process we used a system developed by Lydeard et al. to measure the BIR efficiencies in *ies1*Δ, *ies3*Δ, *pif1*Δ, *rad6*Δ and *cgi121*Δ mutants [74] (Figure 7D). The *rad51*Δ and *rad59*Δ mutant strains served as positive controls [14]. Our results showed that similar to the *rad51*Δ mutant, the *pif1*Δ mutant displayed little BIR efficiency (Figure 7E). In *ies1*Δ, *ies3*Δ and *rad6*Δ mutants, the BIR efficiencies were greatly decreased as also seen in the *rad59*Δ mutant (Figure 7E and 7F). In contrast, the BIR efficiency in the *cgi121*Δ mutant was comparable to that of the wild-type strain (Figure 7F). Taken together, these data indicate that Pif1 is required for Rad51-dependent break-induced-replication, and the INO80 complex and Rad6, but not the KEOPS complex, contribute to this BIR process.

## Discussion

Unlike most other chromosomal loci, eukaryotic telomeres have unique structures attributed to their repetitive DNA sequence and binding proteins [75]. Linear chromosome ends can be recognized as DNA double-stranded breaks and are thus often subjected to repair by non-homologous-end-joining and homologous recombination. It is possible that telomerase-null senescing cells are able to escape the fate of death as telomeres undergo lengthening and repair via homologous recombination. The distinct DNA makeup of Type I and Type II recombinational telomeres allowed us to carry out a genetic screening to identify genes that affect telomere recombination in telomerase-null cells.

Our candidate approach for screening telomere recombination genes had a few shortcomings. In our screening we only covered the 280 known TLM genes, which make up only 5.6% of the ∼5,000 non-essential genes in *S. cerevisiae*. It would be ideal to cover all non-essential genes in our screen. However, such a study would be too massive to undertake since the screening procedures included knocking out *TLC1* in every strain, two to three-weeks passaging cells until they reach senescence and Southern blot experiments for multiple survivors in each mutant (see Figure 1A and Figure 4A). The candidate approach we chose therefore had a strong bias. As a result, we might have missed potential genes that do not affect telomere length, but play important roles in telomere recombination. Another challenge to our screening approach came from the nature of different growth rates of the various mutants. Although we used heterozygous diploid mutants to generate spores of *tlc1*Δ *tlm*Δ double mutants (Table S1), for quite a few mutants we were not able to distinguish between a defect in a survivor pathway and synthetic lethality (Table S1). The third issue that we were not able to resolve was to distinguish between hypo-Type I recombination and hyper-Type II recombination. The decrease of Type I survivor frequency seen in the mutants, such as *rpa14*Δ *tlc1*Δ (Figure 1C) could be caused by either inhibition of Type I recombination or promotion of Type II recombination. In some Type II survivors, the amplified Y'-elements were detected in Southern blot assays (Figure 1C, Figure S1), suggesting that the increase of Type II survivor frequency in these mutants was a result of enhanced Type II recombination rather than inhibited Type I recombination. This model is supported by the observation that in the nine mutants shown in Figure 1C and Figure S1, the emerging events of Type I survivors were significantly reduced, but were not entirely blocked. The fourth issue that we had not taken into consideration during our primary screening was the effect of the initial telomere length of each mutant on the recombination pathways. It was recently proposed that longer telomeres, like those observed in *rif1*Δ and *rif2*Δ mutants could influence the type of recombination pathway used at the telomere [37]. Additionally, it was shown that the *mre11-A470T tlc1*Δ mutant promotes telomere recombination and bypass senescence efficiently because the Type I recombination occurs before growth limitation [76]. Therefore, it would have been more appropriate to perform all of our screening steps starting with *TLC1*/*tlc1Δ TLM*/*tlmΔ* diploids to obtain *tlc1*Δ single and *tlc1*Δ *tlm*Δ double mutants following tetrad dissection. The fifth issue with our screening approach was that we assumed the TLM genes only affect Type I or Type II recombination. Surprisingly, the telomere structure in the *yku* and *pif1* mutants might actually be different from that of a typical Type I or Type II survivor (Figure 3 and Figure 4). Therefore, genes that influence pathway(s) of telomere recombination other than that of Type I or Type II might have been overlooked. The sixth issue with our screen was that we only identified ten novel genes affecting Type I survivor formation (Table 1). This number might be underrepresent the true total because our primary screening was carried out with a relatively stringent criteria and as such we may have overlooked some genes that have minor influences on the frequency Type I survivor emergence.

Although our screening approach had some imperfections, we successfully identified thirty-two TLM genes that influence telomere recombination when overcoming senescence. Ten of these TLM genes affected the emerging frequency of Type I survivors while twenty-two were required for Type II survivor generation. A large portion of 280 TLM genes have not previously been characterized for their roles in telomere function other than the length of the telomeres in these deletion strains was altered. The positive results of our screen provide more direct evidence supporting the idea that some of these uncharacterized TLM genes do affect telomeres [23], [24], [26]. Additionally, telomere recombination is a means by which cells repair defective telomeres and thus the genes involved in telomeric DNA recombination may also play a role in general DNA recombination/repair. Indeed, the TLM genes that affected either Type I or Type II recombination were also required for general DNA recombination (Figure 7A–7C). The annotated functions of the thirty-two genes that we identified point to several pathways that might contribute to telomere maintenance (Table 1 and Table 2). Some of the genes are known for functions like “rRNA processing,” “structural constituent of ribosome,” and “transport and membrane.” These gene products seem unlikely to play a direct role in telomere recombination. In contrast, the Pif1 helicase and the KEOPS complex are involved in “telomere capping and maintenance” [29], [35] and INO80 complex and Rad6 are associated with “chromatin remodeling and modification.” These genes are likely to play direct roles in telomere recombination.

The senescing *pif1*Δ *tlc1*Δ cells did not produce Type I survivors on solid medium (Figure 3C) and the *rad50*Δ *pif1*Δ *tlc1*Δ triple mutant was not able to generate survivors in liquid medium (Figure 3E). These results indicated that Pif1 was required for Type I survivor generation. Interestingly, not all the *rad51*Δ *pif1*Δ *tlc1*Δ triple mutants were able to generate Type II survivors in liquid medium (Figure 3G). Therefore, we favor a model where Pif1 helicase is required for amplification of Y'-elements to form Type I survivors and promotes TG_1–3_ recombination to form Type II survivors (Figure 3). Previous studies have shown that Pif1 takes part in mitochondrial DNA recombination [41]–[43], however, our data are the first to indicate that Pif1 is also involved in telomeric DNA recombination (Figure 3). In the survivors of *pif1*Δ *tlc1*Δ mutants one group exhibited a severely delayed growth phenotype and had a unique telomere structure that differed from the characteristics of either Type I or Type II (Figure 3C). We speculate that these types of survivors require *RAD50* to maintain telomeres since no survivors were recovered in the *pif1*Δ *tlc1*Δ *rad50*Δ triple mutant (Figure 3E). In the future it will be interesting to examine how the short telomeres are maintained in these survivors.

Chromatin remodeling complexes have been shown by others to play roles in DNA repair processes via homologous recombination [77]. However, a causal link between chromatin structure alteration and recombination has not yet been well established. We found that in the absence of active chromatin remodeling by the INO80 complex, telomere Type I recombination was unable to efficiently take place (Figure 2A and 2B), suggesting that the alteration of chromatin structure is a pre-requisite to the Type I recombination process at telomeres. Our results could provide an explanation for the previous observation that *ies3*Δ *est1*Δ cells generated survivors later than the *est1*Δ single mutant [34], as *ies3*Δ *est1*Δ cells likely have a lower efficiency of Type I survivor generation than *est1*Δ cells. *SAP30*, which encodes a subunit of histone deacetylase Rpd3 complex, was also identified in our screening. Deletion of *SAP30* dramatically reduced the emerging rate of the Type I survivors (Table 1), suggesting that the Rpd3 histone deacetylase complex may also inhibit Type I recombination. The SWR1 complex is another chromatin remodeling complex that belongs to the INO80 family of remodeling enzymes. SWR1 and INO80 complexes share four common subunits: Rvb1, Rvb2, Arp4 and Act1 [38]. It will be intriguing to determine if other chromatin remodeling enzymes like SWR1 or histone modification enzymes play roles in telomere recombination.

The KEOPS complex gains its name from “Kinase, Endopeptidase and Other Proteins of small Size” [29], and is comprised of five small proteins (Bud32, Kae1, Pcc1, Gon7 and Cgi121) which form a stable complex *in vitro* and *in vivo* [29], [63], [65], [67]. Bud32 has kinase activity while Kae1 maintains endopeptidase activity [29]. The KEOPS complex or its subunit(s) are involved in several biological processes, to which each KEOPS subunit seems to contribute unequally. Pcc1, Gon7, Kae1 and Bud32, for example, are recruited to several genomic loci and affect gene transcription [65]. Kae1 contributes to faithful chromosome segregation [64] while Bud32, Cgi121, Gon7 regulate cell polarity in bud-site selection [78]. Additionally, Bud32, Kae1 and Pcc1 are essential for a universal tRNA modification called threonyl carbamoyl adenosine (t6A), for which Cgi121 is dispensable [66]. Moreover, all the subunits of the KEOPS complex appear to play roles in telomere uncapping and telomere length regulation [29]. Our screen elucidated a novel function of the KEOPS complex in telomere recombination, as deficiency of any subunit of the KEOPS complex led to the failure in generating Type II survivors in the *tlc1*Δ mutant (Figure 5A). The molecular mechanism by which the KEOPS complex influences telomere recombination remains unclear. A previous study by Downey et al. showed that mutation of the KEOPS complex decreased the amount of single-stranded telomeric DNA in the *cdc13-1* mutant [29]. It is possible that the KEOPS complex facilitates the formation of the telomeric 3′-overhang and promotes recombination of TG-tracts. Coincidently, *SUA5*, a telomeric single-stranded DNA binding protein, is required for both Type II recombination and t6A modification of tRNA [18], [79]. It will be interesting to determine whether *SUA5* is a downstream target of the KEOPS complex and if it functions in the same pathway in regulating telomere recombination. It is possible that Sua5 is a substrate of the Bud32 kinase.

In summary, our screen identified dozens of genes that regulate telomere recombination pathways. Because of the complexity of the recombination process, the molecular mechanisms of telomere recombination remain elusive. Our work not only provides important clues for beginning to understand how telomere recombination is coordinated, but also offers new insights into general DNA repair processes via homologous recombination.

## Materials and Methods

### Yeast strains and plasmids

All strains used in this work are summarized in Table S1 and Table S3. Gene deletions were carried out using standard procedures by genetic cross and homologous recombination. Systematic deletion strains are from EUROSCARF. We constructed CEN plasmids pRS316-PIF1, pRS313-KAE1 and pRS313-BUD32 by inserting fragments (from upstream 1000 bp to downstream 500 bp of genes' open reading frame) into the pRS316 or pRS313 vector. Point mutations were introduced using a site-directed mutagenesis method.

### Cell viability assay

A single colony of the indicated yeast strains was inoculated into 5 ml yeast extract-peptone-dextrose (YPD) medium and grown at 30°C to saturation (OD600 ∼2.5 to 3.0). Then every 24 hours the cell density was measured by spectrometry (OD600) and the cell culture was diluted to the density at OD600 ∼0.02 with fresh YPD medium. This procedure was repeated for up to 14 times, unless the cell density is too low for dilution.

### Single-colony streaking assay

A single colony of the indicated yeast strains was streaked on YPD plate and grown until emergency of single colonies (25 cell divisions) at 30°C. Individual colonies were restreaked repeatedly at least six times to allow survivors to generate.

### Telomere Southern blot

Genomic DNA was prepared from each strain, digested with *Xho*I, separated on 1% gel, transferred to Hybond-N^+^ membrane (GE Healthcare) and then probed with TG_1–3_ telomere-specific probe or Y'-element probe [25]. The *CDC15* probe was ∼263 bp sequence of *CDC15* gene [50].

### General recombination assays

Recombination assays for intrachromosomal and interchromosomal recombination in haploid and diploid strains were performed and recombination rates were determined as previously described [72], [73]. For each mutant, about 2×10^7^ yeast cells were plated on solid selective medium. After growing at 30°C for 2–3 days, about 200 positive colonies would appear on the plate in wild-type haploid strain. Recombination rates were calculated and statistically analyzed by paired two-sample t-test.

### Measurement of break-induced-replication efficiency

Break-induced-replication (BIR) efficiency was measured in a system developed by Lydeard et al [74]. Semi-quantitative PCR was conducted as previously described [74]. PCR products were quantified in Image Quant Software.

## Supporting Information

Figure S1 Southern blot analysis of survivor types in *tlc1*Δ tlmΔ double mutants. The *tlm*Δ *tlc1*Δ double mutants were generated through tetrad dissection from heterozygous diploids with one copy of *TLM* gene and *TLC1* deleted. The mutants tested and shown are (A) *rif1*Δ *tlc1*Δ, (B) *rif2*Δ *tlc1*Δ, (C) *sap30*Δ *tlc1*Δ, (D) *rpb9*Δ *tlc1*Δ, (E) *soh1*Δ *tlc1*Δ,Δ(F) *rrp8*Δ *tlc1*Δ, (G) *rps16b*Δ *tlc1*Δ and (H) *gup1*Δ *tlc1*Δ. Fifty independent colonies of each mutant were randomly selected and passaged on solid plates, and the telomere structures of survivors were examined by Southern blot using a TG probe. The triangles (▾) indicate Type I survivors, while the others are Type II survivors. The frequencies of Type II survivors were calculated and summarized in Table 1.
(TIF)

Figure S2 Southern blot analysis of telomere lengths at several passages around survivor emerging point in *tlc1*Δ single and *tlc1*Δ*tlm*Δ double mutants. Heterozygous diploid strains with one copy of *TLM* gene and *TLC1* deleted were dissected and then these isogenic spores from the same crosses were subjected to survivor analysis. Around the survivor emerging point, for each pair of *tlc1*Δ and *tlm*Δ *tlc1*Δhaploid strains originated from the same cross, cells in four successive passages were collected, and their genomic DNAs was extracted and subjected to Southern blot analysis using a TG probe (upper panels). Telomere lengths were quantified by Image Quant software and plotted (lower panels). The mutants tested and shown are (A) *rpa14*Δ *tlc1*Δ, (B) *rif1*Δ *tlc1*Δ, (C)*rif2*Δ *tlc1*Δ, (D) *sap30*Δ *tlc1*Δ, (E) *rpb9*Δ *tlc1*Δ,(F) *soh1*Δ *tlc1*Δ, (G) *rrp8*Δ *tlc1*Δ, (H) *rps16b*Δ *tlc1*Δ, (I) *gup1*Δ *tlc1*Δ and (J) *ino80*Δ *tlc1*Δ. The isogenic strains are labeled on top and the passage numbers are labeled under each strain. The full image of Southern blot membrane for *rpa14*Δ *tlc1*Δ and *tlc1*Δ control is shown in (A) while only partial image of Southern blot membrane, i.e. the terminal-restriction-fragment image, for other mutants are shown in panels (B) through (J).
(TIF)

Figure S3 Southern blot analysis of survivor types in *tlc1*Δ that were also lacking a member of the INO80 complex. A *tlc1*Δ single deletion mutant was generated through tetrad dissection from heterozygous diploids with one copy of *TLC1* and *IES4* deleted. The *ies1*Δ *tlc1*Δ, *ies4*Δ *tlc1*Δ, *ies5*Δ *tlc1*Δ and *nhp10*Δ *tlc1*Δ double mutants were generated through tetrad dissection from heterozygous diploids with one copy of Ino80 complex subunits gene and *TLC1* deleted. Results from the mutants (A) *tlc1*Δ, (B) *ies1*Δ *tlc1*Δ, (C) *ies4*Δ *tlc1*Δ, (D) *ies5*Δ *tlc1*Δ and (E) *nhp10*Δ *tlc1*Δ are shown. Fifty independent colonies of each mutant were randomly selected and passaged on solid plates, and the telomere structures of survivors were examined by Southern blot using a TG probe. The triangles (▾) indicate Type I survivors, while others are Type II survivors. The frequencies of Type II survivors were calculated and summarized in Figure 2B (column of “Spores from tetrad dissection”).
(TIF)

Figure S4 The effect of thirteen DNA helicase genes on survivor formation. Thirteen DNA helicase genes were knocked out in a *TLC1* deletion mutant. These double mutants were either passaged on plates (A) or serially cultured in liquid medium (B) until survivors generated. The telomere structures of survivors were examined by Southern blot assay. (A) On plates, only the *pif1*Δ *tlc1*Δ mutant could not form Type I survivors. (B) In liquid cultures, the *sgs1*Δ *tlc1*Δ mutant could only form Type I survivors.
(TIF)

Figure S5 Cell viability assay of rad6Δ and its downstream target gene mutants. The heterozygous diploid *TLC1*/*tlc1*Δ *RAD18*/*rad18*Δ *BRE1*/*bre1*Δ *UBR1*/*ubr1*Δ mutant was sporulated and tetrads were dissected. One spore of *tlc1*Δ single mutant and three spores of each genotype of (A) *ubr1*Δ *tlc1*Δ, (B) *rad18*Δ *tlc1*Δ, (C) *bre1*Δ *ubr1*Δ *tlc1*Δ, (D) *bre1*Δ *rad18*Δ *tlc1*Δ, (E) u*br1*Δ *rad18*Δ *tlc1*Δ and (F) *bre1*Δ *ubr*1Δ *rad18*Δ *tlc1*Δ were subjected to cell viability assay and the corresponding strains are indicated in each panel.
(TIF)

Figure S6 Representative gels of the BIR repair product in wild type, *rad51*Δ, *rad59*Δ, *ies1*Δ, *ies3*Δ, *pif1*Δ, *rad6*Δ and *cgi121*Δ cells. Cells were harvested at 0, 1, 3, 6, 8, 10, 12 and 24 hr after HO induction, then the genomic DNA was extracted and subjected to semi-quantitative PCR. The BIR repair products were labeled as “*CAN1*” and reference PCR products of the *FLO9* locus were displayed as loading controls.
(TIF)

Table S1 Complete list of 280 TLM genes.
(XLS)

Table S2 List of screened-out genes which had been reported in DNA repair and recombination.
(DOC)

Table S3 Yeast strains used in this study.
(DOC)
