# Supplementary figures and images for: Telomerase-Null Survivor Screening Identifies Novel Telomere Recombination Regulators
Source: PLoS Genet. 2013 Jan 17;9(1):e1003208. doi: 10.1371/journal.pgen.1003208 (PMC3547846; doi:10.1371/journal.pgen.1003208)

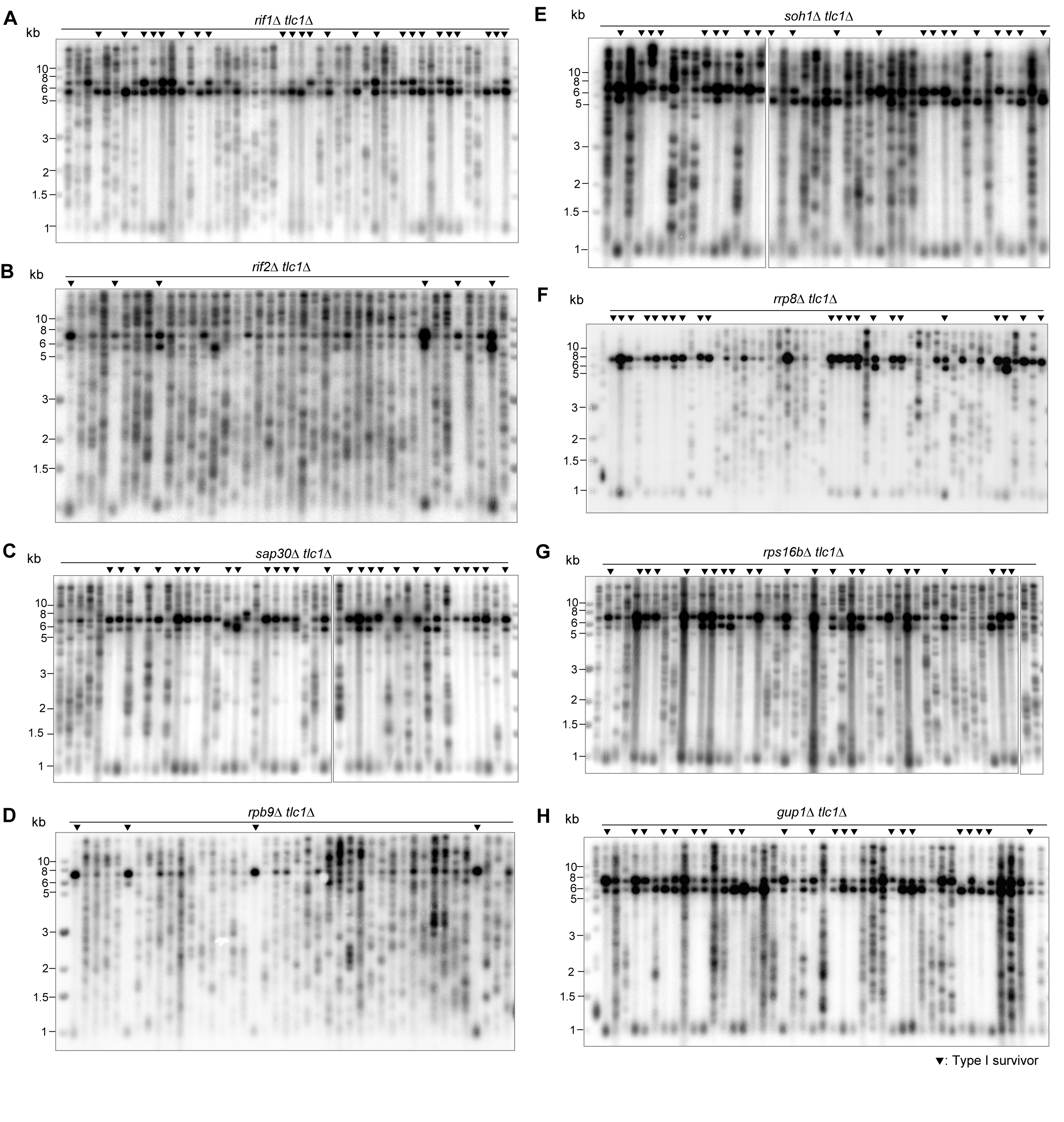

Supplement: Figure S1 — Southern blot analysis of survivor types in tlc1Δ tlmΔ double mutants. The tlmΔ tlc1Δ double mutants were generated through tetrad dissection from heterozygous diploids with one copy of TLM gene and TLC1 deleted. The mutants tested and shown are (A) rif1Δ tlc1Δ, (B) rif2Δ tlc1Δ, (C) sap30Δ tlc1Δ, (D) rpb9Δ tlc1Δ, (E) soh1Δ tlc1Δ,Δ(F) rrp8Δ tlc1Δ, (G) rps16bΔ tlc1Δ and (H) gup1Δ tlc1Δ. Fifty independent colonies of each mutant were randomly selected and passaged on solid plates, and the telomere structures of survivors were examined by Southern blot using a TG probe. The triangles (▾) indicate Type I survivors, while the others are Type II survivors. The frequencies of Type II survivors were calculated and summarized in Table 1. (TIF) [file pgen.1003208.s001.tif]

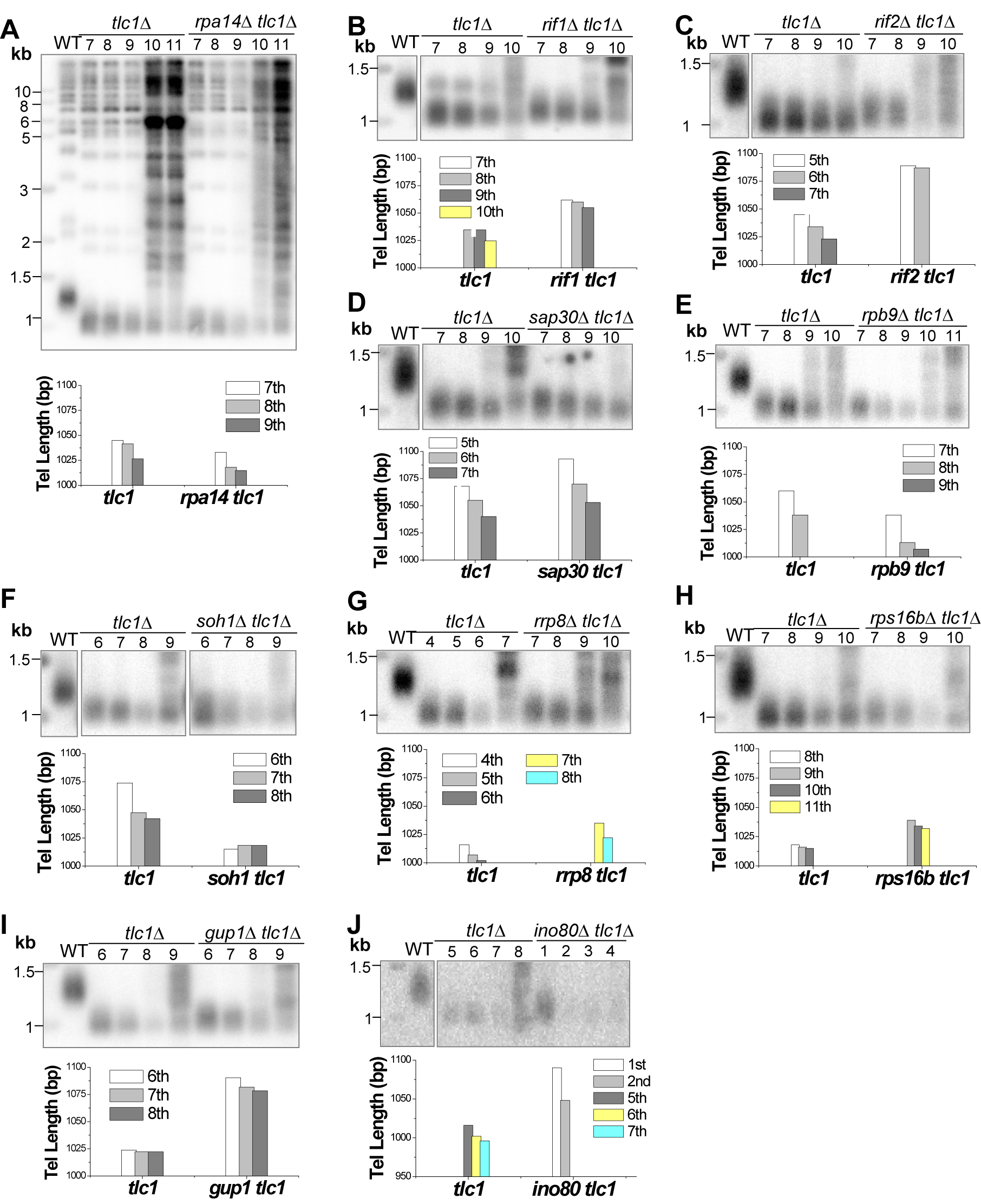

Supplement: Figure S2 — Southern blot analysis of telomere lengths at several passages around survivor emerging point in tlc1Δ single and tlc1ΔtlmΔ double mutants. Heterozygous diploid strains with one copy of TLM gene and TLC1 deleted were dissected and then these isogenic spores from the same crosses were subjected to survivor analysis. Around the survivor emerging point, for each pair of tlc1Δ and tlmΔ tlc1Δhaploid strains originated from the same cross, cells in four successive passages were collected, and their genomic DNAs was extracted and subjected to Southern blot analysis using a TG probe (upper panels). Telomere lengths were quantified by Image Quant software and plotted (lower panels). The mutants tested and shown are (A) rpa14Δ tlc1Δ, (B) rif1Δ tlc1Δ, (C)rif2Δ tlc1Δ, (D) sap30Δ tlc1Δ, (E) rpb9Δ tlc1Δ,(F) soh1Δ tlc1Δ, (G) rrp8Δ tlc1Δ, (H) rps16bΔ tlc1Δ, (I) gup1Δ tlc1Δ and (J) ino80Δ tlc1Δ. The isogenic strains are labeled on top and the passage numbers are labeled under each strain. The full image of Southern blot membrane for rpa14Δ tlc1Δ and tlc1Δ control is shown in (A) while only partial image of Southern blot membrane, i.e. the terminal-restriction-fragment image, for other mutants are shown in panels (B) through (J). (TIF) [file pgen.1003208.s002.tif]

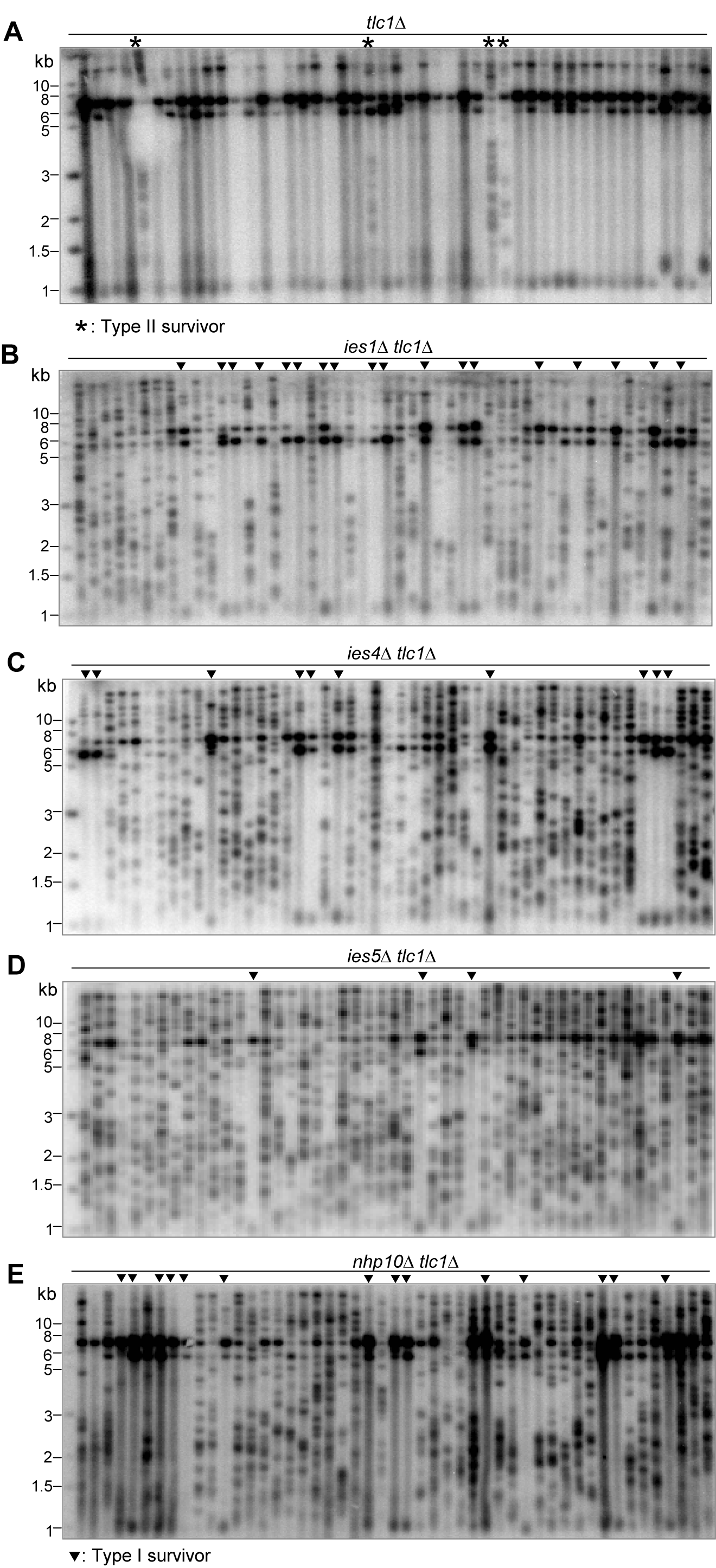

Supplement: Figure S3 — Southern blot analysis of survivor types in tlc1Δ that were also lacking a member of the INO80 complex. A tlc1Δ single deletion mutant was generated through tetrad dissection from heterozygous diploids with one copy of TLC1 and IES4 deleted. The ies1Δ tlc1Δ, ies4Δ tlc1Δ, ies5Δ tlc1Δ and nhp10Δ tlc1Δ double mutants were generated through tetrad dissection from heterozygous diploids with one copy of Ino80 complex subunits gene and TLC1 deleted. Results from the mutants (A) tlc1Δ, (B) ies1Δ tlc1Δ, (C) ies4Δ tlc1Δ, (D) ies5Δ tlc1Δ and (E) nhp10Δ tlc1Δ are shown. Fifty independent colonies of each mutant were randomly selected and passaged on solid plates, and the telomere structures of survivors were examined by Southern blot using a TG probe. The triangles (▾) indicate Type I survivors, while others are Type II survivors. The frequencies of Type II survivors were calculated and summarized in Figure 2B (column of “Spores from tetrad dissection”). (TIF) [file pgen.1003208.s003.tif]

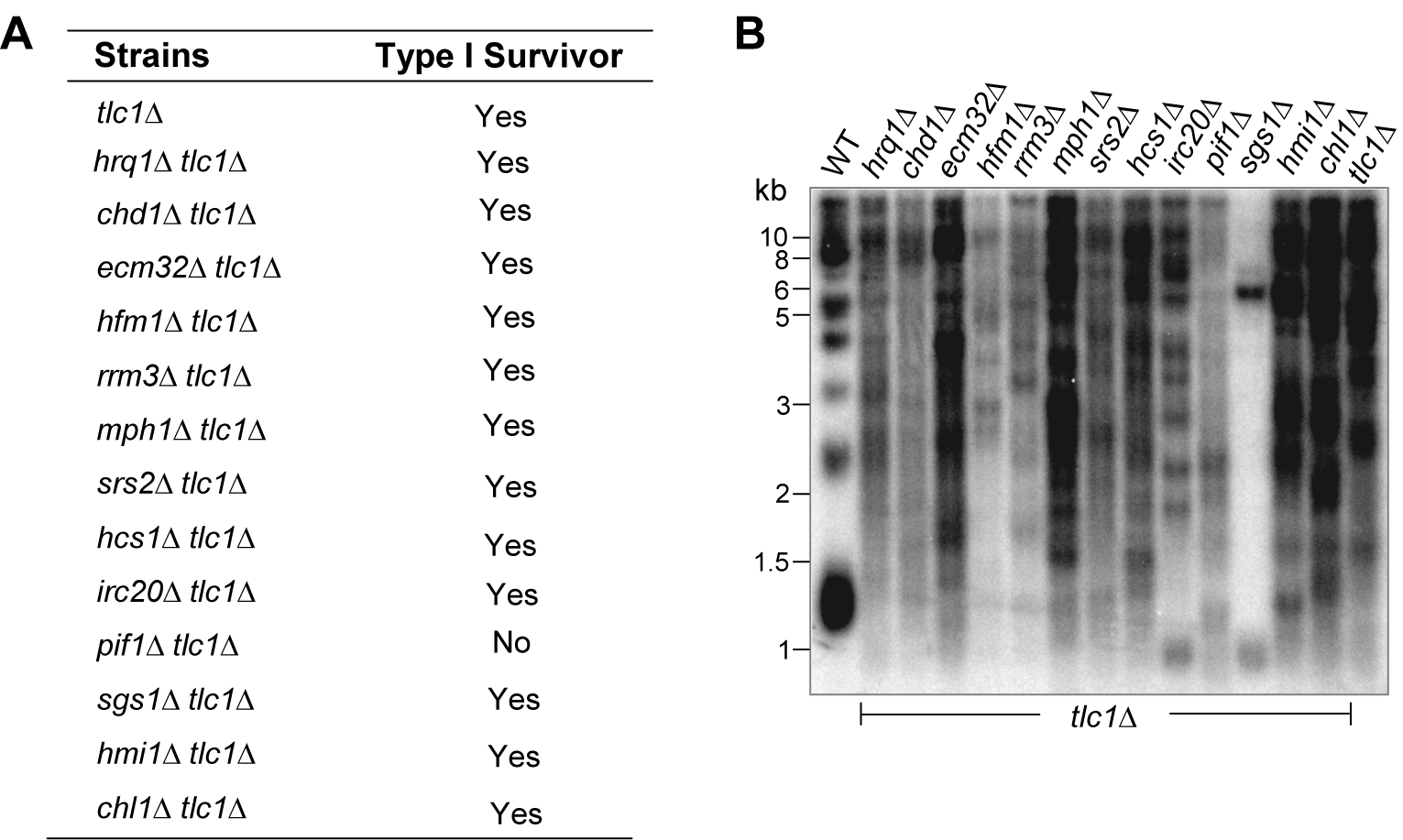

Supplement: Figure S4 — The effect of thirteen DNA helicase genes on survivor formation. Thirteen DNA helicase genes were knocked out in a TLC1 deletion mutant. These double mutants were either passaged on plates (A) or serially cultured in liquid medium (B) until survivors generated. The telomere structures of survivors were examined by Southern blot assay. (A) On plates, only the pif1Δ tlc1Δ mutant could not form Type I survivors. (B) In liquid cultures, the sgs1Δ tlc1Δ mutant could only form Type I survivors. (TIF) [file pgen.1003208.s004.tif]

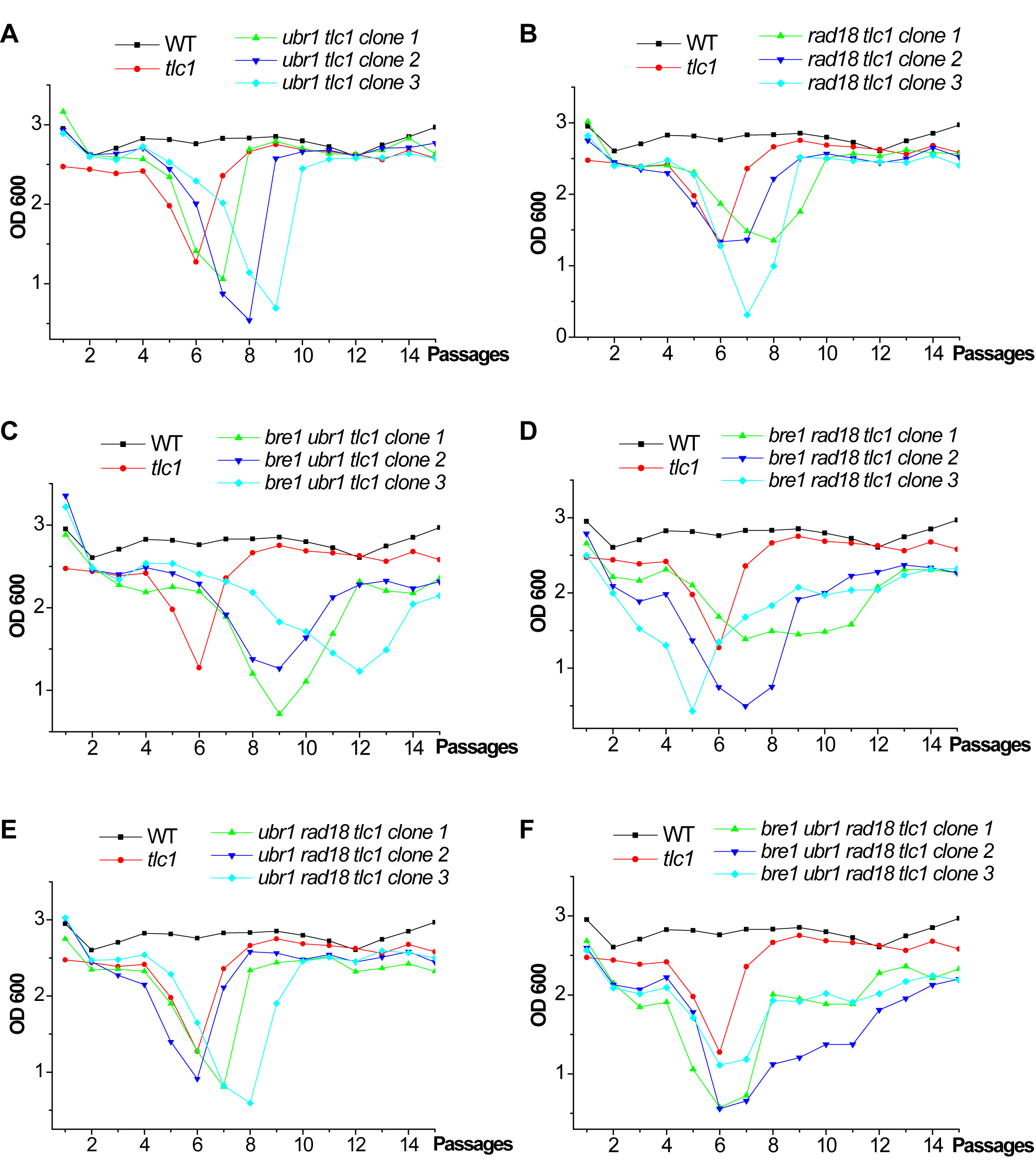

Supplement: Figure S5 — Cell viability assay of rad6Δ and its downstream target gene mutants. The heterozygous diploid TLC1/tlc1Δ RAD18/rad18Δ BRE1/bre1Δ UBR1/ubr1Δ mutant was sporulated and tetrads were dissected. One spore of tlc1Δ single mutant and three spores of each genotype of (A) ubr1Δ tlc1Δ, (B) rad18Δ tlc1Δ, (C) bre1Δ ubr1Δ tlc1Δ, (D) bre1Δ rad18Δ tlc1Δ, (E) ubr1Δ rad18Δ tlc1Δ and (F) bre1Δ ubr1Δ rad18Δ tlc1Δ were subjected to cell viability assay and the corresponding strains are indicated in each panel. (TIF) [file pgen.1003208.s005.tif]

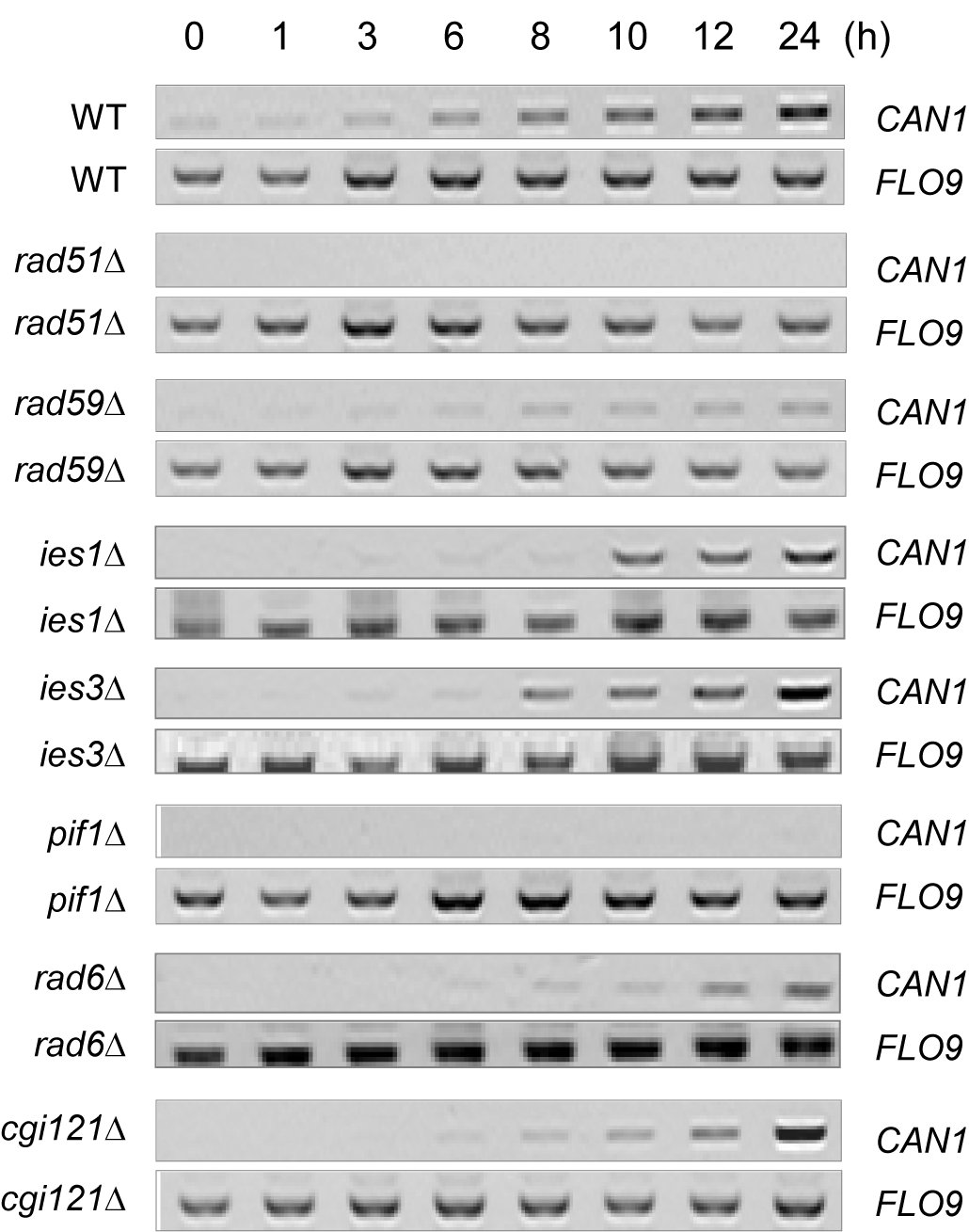

Supplement: Figure S6 — Representative gels of the BIR repair product in wild type, rad51Δ, rad59Δ, ies1Δ, ies3Δ, pif1Δ, rad6Δ and cgi121Δ cells. Cells were harvested at 0, 1, 3, 6, 8, 10, 12 and 24 hr after HO induction, then the genomic DNA was extracted and subjected to semi-quantitative PCR. The BIR repair products were labeled as “CAN1” and reference PCR products of the FLO9 locus were displayed as loading controls. (TIF) [file pgen.1003208.s006.tif]
