# Supplementary material for: Telomerase-Null Survivor Screening Identifies Novel Telomere Recombination Regulators
Source: PLoS Genet. 2013 Jan 17;9(1):e1003208. doi: 10.1371/journal.pgen.1003208 (PMC3547846; doi:10.1371/journal.pgen.1003208)
Supplement: Table S2 — List of screened-out genes which had been reported in DNA repair and recombination. (DOC) [file pgen.1003208.s008.doc]

**Table S2**. List of screened-out genes which had been reported in DNA repair and recombination

| **Gene** | **Survivor Type** | **Genome-wide Screen** | | | **Individual**  **Study** | **References** |
| --- | --- | --- | --- | --- | --- | --- |
|  |  | **ROS^a^** | **LOH^b^** | **SSA^c^** |  |  |
| ***CBC2*** | II | **×**^d^ |  | **×** |  | [1-2] |
| ***CDH1*** | II |  | **×** |  |  | [3] |
| ***GUP1*** | I |  |  | **×** |  | [2] |
| ***IES3*** | I |  | **×** |  |  | [3] |
| ***INO80*** | I |  |  |  | **×** | [4] |
| ***XRN1*** | II |  |  |  | **×** | [5-6] |
| ***MET7*** | II | **×** |  |  |  | [1] |
| ***PIF1*** | I |  |  | **×** | **×** | [2,7] |
| ***RAD6*** | II | **×** | **×** |  | **×** | [1,3,8] |
| ***RPA14*** | I |  | **×** | **×** |  | [2-3] |
| ***RPB9*** | I | **×** |  |  |  | [1] |
| ***RPL13B*** | II |  |  |  | **×** | [9] |
| ***RRP8*** | I |  |  | **×** |  | [2] |
| ***SAP30*** | I |  |  | **×** |  | [2] |
| ***SLX8*** | II | **×** | **×** |  | **×** | [1,3,10-11] |

^a^ROS: DNA repair in response to highly reactive oxygen species.

^b^LOH: Loss of heterozygosity.

^c^SSA: Single strand annealing.

^d^This gene had been reported in the study of the shown aspect.

1. Westmoreland TJ, Wickramasekara SM, Guo AY, Selim AL, Winsor TS, et al. (2009) Comparative genome-wide screening identifies a conserved doxorubicin repair network that is diploid specific in Saccharomyces cerevisiae. PLoS One 4: e5830.

2. Li F, Dong J, Pan X, Oum JH, Boeke JD, et al. (2008) Microarray-based genetic screen defines SAW1, a gene required for Rad1/Rad10-dependent processing of recombination intermediates. Mol Cell 30: 325-335.

3. Andersen MP, Nelson ZW, Hetrick ED, Gottschling DE (2008) A genetic screen for increased loss of heterozygosity in Saccharomyces cerevisiae. Genetics 179: 1179-1195.

4. Tsukuda T, Fleming AB, Nickoloff JA, Osley MA (2005) Chromatin remodelling at a DNA double-strand break site in Saccharomyces cerevisiae. Nature 438: 379-383.

5. Johnson AW, Kolodner RD (1991) Strand exchange protein 1 from Saccharomyces cerevisiae. A novel multifunctional protein that contains DNA strand exchange and exonuclease activities. J Biol Chem 266: 14046-14054.

6. Tishkoff DX, Johnson AW, Kolodner RD (1991) Molecular and genetic analysis of the gene encoding the Saccharomyces cerevisiae strand exchange protein Sep1. Mol Cell Biol 11: 2593-2608.

7. Wagner M, Price G, Rothstein R (2006) The absence of Top3 reveals an interaction between the Sgs1 and Pif1 DNA helicases in Saccharomyces cerevisiae. Genetics 174: 555-573.

8. Game JC, Chernikova SB (2009) The role of RAD6 in recombinational repair, checkpoints and meiosis via histone modification. DNA Repair (Amst) 8: 470-482.

9. Smith S, Hwang JY, Banerjee S, Majeed A, Gupta A, et al. (2004) Mutator genes for suppression of gross chromosomal rearrangements identified by a genome-wide screening in Saccharomyces cerevisiae. Proc Natl Acad Sci U S A 101: 9039-9044.

10. Zhang C, Roberts TM, Yang J, Desai R, Brown GW (2006) Suppression of genomic instability by SLX5 and SLX8 in Saccharomyces cerevisiae. DNA Repair (Amst) 5: 336-346.

11. Burgess RC, Rahman S, Lisby M, Rothstein R, Zhao X (2007) The Slx5-Slx8 complex affects sumoylation of DNA repair proteins and negatively regulates recombination. Mol Cell Biol 27: 6153-6162.
