# Supplementary material for: Telomerase-Null Survivor Screening Identifies Novel Telomere Recombination Regulators
Source: PLoS Genet. 2013 Jan 17;9(1):e1003208. doi: 10.1371/journal.pgen.1003208 (PMC3547846; doi:10.1371/journal.pgen.1003208)
Supplement: Table S3 — Yeast strains used in this study. (DOC) [file pgen.1003208.s009.doc]

**Table S3. Yeast strains used in this study**

| **Strain** | **Relevant genotype** | **Source** |
| --- | --- | --- |
| BY4741 | *MATa his3Δ1 leu2Δ0 met15Δ0 ura3Δ0* | Euroscarf |
| BY4742 | *MATΔ his3Δ1 leu2Δ0 lys2Δ0 ura3Δ0* | Euroscarf |
| BY4743 | *MATa/Δ his3Δ1/his3Δ1 leu2Δ0/leu2Δ0 lys2Δ0/LYS2 MET15/met15Δ0 ura3Δ0/ura3Δ0* | Euroscarf |
| YPH499 | *MATa* *ura3-52 lys2-801_amber ade2-101_ochre trp1-Δ63 his3-Δ200 leu2-Δ1* | [1] |
| YPH501 | *MATa/Δ* *ura3-52/ura3-52 lys2-801_amber/lys2-801_amber ade2-101_ochre/ade2-101_ochre trp1-Δ63/trp1-Δ63 his3-Δ200/his3-Δ200 leu2-Δ1/leu2-Δ1* | [1] |
| HKY660-2B | *MATΔ* *leu2*-*EcoRI*::*URA3*::*leu2*-*BstEII* *ade2-1* *can1-100* *his3-11,15 trp1-1 ura3-1* | [2] |
| HKY716-5D | *MATa* *leu2-ecoRI-bstEII ade2-1 can1-100 his3-11,15 trp1-1 ura3-1* | [2] |
| HKY666-1A | *MATΔ* *leu2-ecoRI ade2-1 can1-100 his3-11,15 trp1-1 ura3-1* | [2] |
| HKY679-2C | *MATa* *leu2-bstEII ade2-1 can1-100 his3-11,15 trp1-1 ura3-1* | [2] |
| JRL346 | *mata::HOcsDEL::hisG ura3 DEL851 trp1DEL63 sup53DEL::leu2DEL::NATMX hmlDEL::hisG hmrDEL::ADE3 ade3::GAL10::HO can1,1-1446::HOcs::HPH::DEL AVT2 ykl215c::leu2::hisG::can1DEL1-289* | [3] |
| JRL636 | *mata::HOcsDEL::hisG ura3 DEL851 trp1DEL63 sup53DEL::leu2DEL::NATMX hmlDEL::hisG hmrDEL::ADE3 ade3::GAL10::HO can1,1-1446::HOcs::HPH::DEL AVT2 ykl215c::leu2::hisG::can1DEL1-289 rad51::URA3* | [3] |
| HY001 | *mata::HOcsDEL::hisG ura3 DEL851 trp1DEL63 sup53DEL::leu2DEL::NATMX hmlDEL::hisG hmrDEL::ADE3 ade3::GAL10::HO can1,1-1446::HOcs::HPH::DEL AVT2 ykl215c::leu2::hisG::can1DEL1-289 rad59::URA3* | this work |
| HY002 | *mata::HOcsDEL::hisG ura3 DEL851 trp1DEL63 sup53DEL::leu2DEL::NATMX hmlDEL::hisG hmrDEL::ADE3 ade3::GAL10::HO can1,1-1446::HOcs::HPH::DEL AVT2 ykl215c::leu2::hisG::can1DEL1-289 pif1::URA3* | this work |
| HY003 | *mata::HOcsDEL::hisG ura3 DEL851 trp1DEL63 sup53DEL::leu2DEL::NATMX hmlDEL::hisG hmrDEL::ADE3 ade3::GAL10::HO can1,1-1446::HOcs::HPH::DEL AVT2 ykl215c::leu2::hisG::can1DEL1-289 cgi121::URA3* | this work |
| LNN001 | BY4743 *RAD6/rad6*Δ*::URA3 Rad51/rad51*Δ*::HIS3 TLC1/tlc1*Δ*::LEU2* | this work |
| LNN002 | BY4743 *BRE1/bre1*Δ*::URA3 UBR1/ubr1*Δ*::KanMX4 RAD18/rad18*Δ*::HIS3 TLC1/tlc1*Δ*::LEU2* | this work |
| LNN003 | BY4742 *ies1*Δ*::KanMX4* | Euroscarf |
| LNN004 | BY4742 *ies3*Δ*::KanMX4* | Euroscarf |
| LNN005 | BY4742 *ies4*Δ*::KanMX4* | Euroscarf |
| LNN006 | BY4742 *ies5*Δ*::KanMX4* | Euroscarf |
| LNN007 | BY4742 *nhp10*Δ*::KanMX4* | Euroscarf |
| THB001 | BY4743 *PIF1*/*pif1*Δ::*HIS3* *TLC1*/*tlc1*Δ::*LEU2* | this work |
| THB002 | BY4743 *PIF1*/*pif1*Δ::*HIS3* *TLC1*/*tlc1*Δ::*LEU2*/*CEN* pRS316*-pif1*-K264A | this work |
| THB003 | BY4743 *PIF1*/*pif1*Δ:: *HIS3* *TLC1*/*tlc1*Δ::*LEU2 RAD50*/*rad50*Δ::*URA3* | this work |
| THB004 | BY4743 *PIF1*/*pif1*Δ::*HIS3* *TLC1*/*tlc1*Δ::*LEU2 SGS1* /*sgs1*Δ::*URA3* | this work |
| THB005 | BY4743 *KAE1*/kae1Δ*KanMX4* *TLC1*/*tlc1*Δ::*LEU2* | this work |
| THB006 | BY4743 *CGI121*/*cgi121*Δ::*URA3* *TLC1*/*tlc1*Δ::*LEU2* | this work |
| THB007 | BY4743 *BUD32*/*bud32*Δ::*URA3 TLC1*/*tlc1*Δ::*LEU2* | this work |
| THB008 | BY4743 *GON7*/*gon7*Δ::*HIS3 TLC1*/*tlc1*Δ::*LEU2* | this work |
| THB009 | BY4743 *PCC1*/*pcc1*Δ::*HIS3 TLC1*/*tlc1*Δ::*LEU2* | this work |
| THB010 | BY4743 *KAE1*/*kae1*Δ*anMX4* *TLC1*/*tlc1*Δ::*LEU2*/*CEN* pRS313*-kae1*-E213R | this work |
| THB011 | BY4743 *KAE1*/*kae1*Δ*anMX4* *TLC1*/*tlc1*Δ::*LEU2*/*CEN* pRS313*-kae1*-E292R | this work |
| THB012 | BY4743 *KAE1*/*kae1*Δ*KanMX4* *TLC1*/*tlc1*Δ::*LEU2*/*CEN* pRS313*-kae1*-E295K | this work |
| THB013 | BY4743 *BUD32*/*bud32*Δ::*URA3 TLC1*/*tlc1*Δ::*LEU2*/*CEN* pRS313*-bud32*-K52A | this work |
| THB014 | BY4743 *BUD32*/*bud32*Δ::*URA3 TLC1*/*tlc1*Δ::*LEU2*/*CEN* pRS313*-bud32*-N166A | this work |
| THB015 | BY4743 *PIF1*/*pif1*Δ:: *HIS3* *TLC1*/*tlc1*Δ::*LEU2 RAD51*/*rad51*Δ::*URA3* | this work |
| HY004 | BY4743 *CGI121*/*cgi121*Δ::*URA3* *TLC1*/*tlc1*Δ::*LEU2 RAD51*/*rad51*Δ:: *HIS3* | this work |
| HY005 | BY4743 *CGI121*/*cgi121*Δ::*URA3* *TLC1*/*tlc1*Δ::*LEU2 RAD50*/*rad50*Δ:: *HIS3* | this work |

**References:**

1. Sikorski RS, Hieter P (1989) A system of shuttle vectors and yeast host strains designed for efficient manipulation of DNA in Saccharomyces cerevisiae. Genetics 122: 19-27.

2. Aguilera A, Klein HL (1988) Genetic control of intrachromosomal recombination in Saccharomyces cerevisiae. I. Isolation and genetic characterization of hyper-recombination mutations. Genetics 119: 779-790.

3. Lydeard JR, Jain S, Yamaguchi M, Haber JE (2007) Break-induced replication and telomerase-independent telomere maintenance require Pol32. Nature 448: 820-823.
